# Detection of PRMT1 inhibitors with stopped flow fluorescence

**DOI:** 10.1038/s41392-018-0009-6

**Published:** 2018-03-09

**Authors:** Kun Qian, Hao Hu, Hui Xu, Y George Zheng

**Affiliations:** 1 Department of Pharmaceutical and Biomedical Sciences, University of Georgia, Athens, GA 30602 USA; 2 Present Address: The Chemical Proteomics Center and State Key Laboratory of Drug Research, Shanghai Institute of Materia Medica, Chinese Academy of Sciences, Shanghai, 201203 China

## Abstract

Protein arginine methyltransferases (PRMTs) are crucial epigenetic regulators in eukaryotic organisms that serve as histone writers for chromatin remodeling. PRMTs also methylate a variety of non-histone protein substrates to modulate their function and activity. The development of potent PRMT inhibitors has become an emerging and imperative research area in the drug discovery field to provide novel therapeutic agents for treating diseases and as tools to investigate the biological functions of PRMTs. PRMT1 is the major type I enzyme that catalyzes the formation of asymmetric dimethyl arginine, and PRMT1 plays important regulatory roles in signal transduction, transcriptional activation, RNA splicing, and DNA repair. Aberrant expression of PRMT1 is found in many types of cancers, pulmonary diseases, cardiovascular disease, diabetes, and renal diseases. PRMT1 is a highly promising target for therapeutic development. We created a stopped flow fluorescence-based assay for PRMT1 inhibitor detection and characterization that has the advantages of being homogeneous, nonradioactive, and mix-and-measure in nature, allowing for continuous measurement of the methylation reaction and its inhibition. To our knowledge, this is the first continuous assay for PRMT1 reaction detection and inhibitor characterization. The approach is not only capable of quantitatively determining the potency (IC_50_) of PRMT1 inhibitors but can also distinguish cofactor-competitive inhibitors, substrate-competitive inhibitors, and mixed-type inhibitors.

## Biochemistry: Innovative strategy to detect enzyme inhibition

Protein arginine methyltransferases (PRMTs) catalyzes the transfer of methyl groups to arginine residues in cellular proteins. The resultant arginine methylation plays important regulatory roles for signal transductions that are related to various diseases, especially cancers. Qian and colleagues developed a novel method called stopped flow fluorescence assay to sensitively measure the PRMT-mediated methylation. Using fluorescently-labeled peptide substrates, they were able to spectroscopically track the progress of arginine methylation throughout the reaction course. Interestingly, they found that different PRMT inhibitors impact on the fluorescent time course in divergent ways. PRMT1-catalyzed methylation follows two kinetic phases, the inhibition pattern of Phase I can distinguish the mechanism of action while that of Phase II can determine the potency of the tested inhibitors. This time-resolved enzyme assay strategy provides a novel means to enzyme inhibitor discovery and mode-of-action elucidation.

## Introduction

Protein arginine methylation is a type of universal posttranslational modification (PTM) that plays significant biological roles in eukaryotic organisms.^1^ Thus far, nine protein arginine methyltransferases (PRMTs) have been found in mammalian cells,^2^ which are classified into three types: type I, type II and type III PRMTs. Type I enzymes (PRMT1, −2, −3, −4, −6, and −8) convert arginine residues to monomethyl arginine (MMA) and further modify them to asymmetric dimethyl arginine (ADMA); type II enzymes (PRMT5 and PRMT9) produce MMA and symmetric dimethyl arginine (SDMA); and PRMT7 is the only type III enzyme that generates MMA. The global arginine levels in mouse embryo fibroblast (MEF) cells have been found to be 1500:3:2:1 for Arg:ADMA:MMA:SDMA, and PRMT1 is the major type I enzyme, accounting for 50% of ADMA formation.^3,4^ During PRMT catalysis, one or two hydrogen atom(s) on the ω-N^G^ of arginine substrate is (are) replaced by the methyl group from *S*-adenosylmethionine (SAM or AdoMet), generating methylated arginine and leaving *S*-adenosyl homocysteine (SAH or AdoHcy) as the side product.^2^ PRMTs methylate numerous protein substrates in the nucleus, cytoplasm, and membranes.^1^ Studies have revealed diverse roles of PRMTs in signal transduction, transcriptional coactivation, RNA splicing and DNA repair, while its other functions remain unclear.^5^ Moreover, dysregulation or aberrant expression of PRMTs is associated with various pathological conditions. For instance, PRMT1 is overexpressed or aberrantly expressed in breast, prostate, lung, colon, and bladder cancers, and leukemia.^5,6^ It is also upregulated in pulmonary diseases such as pulmonary fibrosis, pulmonary hypertension, chronic obstructive pulmonary disease (COPD), and asthma.^7^ Further, PRMT1 plays regulatory roles in cardiovascular disease, diabetes, and renal diseases.^8^ Therefore, the development of PRMT inhibitors has emerged as an imperative task to provide novel therapeutic agents to treat diseases and to find chemical probes to investigate the biological functions of PRMTs.^7,9,10^

In the past decade, both academic and industrial laboratories have invested effort to discover and develop PRMT inhibitors possessing adequate potency and isoform selectivity.^7,10–13^ The discovery of PRMT inhibitors relies on efficient and effective biochemical assays for measuring the methyltransferase activity of PRMTs and for characterizing the mechanism of inhibitors.^14–23^ Radiometric assays represent the gold standard for biochemically measuring the methyltransferase activity of PRMTs due to their high sensitivity and reliability.^7^ In this type of assay, the radioisotope-labeled methyl group of [^3^H]-SAM or [^14^C]-SAM is transferred to a peptide or protein substrate during the enzymatic reaction.^7^ The products are then separated from unreacted SAM and quantified by autoradiography or liquid scintillation counting. Among these radiometric assays, scintillation proximity assay (SPA) is a mix-and-measure procedure not requiring product separation, in which the signal is induced by the micrometer proximity between biotinylated substrates containing ^3^H labeled methyl groups and streptavidin-coated scintillatants, while the excess SAM molecules in the solution are not within this distance and therefore do not produce signals.^24^ This assay format can be applied for the high-throughput screening of library compounds to discover PRMT inhibitors.^24^ However, one key drawback of this type of assay is the involvement of radioactive reagents, which requires strict environmental safety regulation. Another type of assay is antibody-based, exemplified by the enzyme-linked immunosorbent assay (ELISA), in which a methylarginine-specific antibody is used to recognize reaction products and a secondary antibody is used as probe for signal detection.^7^ A few enzymatically coupled assays have also been developed to detect the generation of the side product SAH for measuring PRMT activity; these assays often convert SAH into chemical derivatives bearing colorimetric, fluorescent, or luminescent properties for spectroscopic signal detection.^25–30^ These assays are nonradioactive and robust; however, introducing additional components in the assay can potentially complicate the assay results. Especially for inhibitor screening, the coupling chemical components might interact with inhibitors and lead to false positives. Furthermore, due to the variability and complexity of detection methods, all the above assays require the methyltransferase reaction to be quenched at specific time points, after which the products are processed or converted into other chemical species for signal generation. In these scenarios, it is not possible to monitor the arginine methylation reaction progression in situ. In this paper, we have developed a stopped flow fluorescent assay to detect and characterize PRMT1 inhibitors, which possesses the advantages of being nonradioactive and homogeneous. The assay can be implemented through a simple mix-and-measure procedure, and products can be detected continuously. To the best of our knowledge, this is the first continuous assay for PRMT reaction detection and inhibitor characterization.

## Results and discussion

### Fluorescent changes of fluorescein-labeled histone H4 peptide during PRMT1 catalysis

**Fig. 1:**
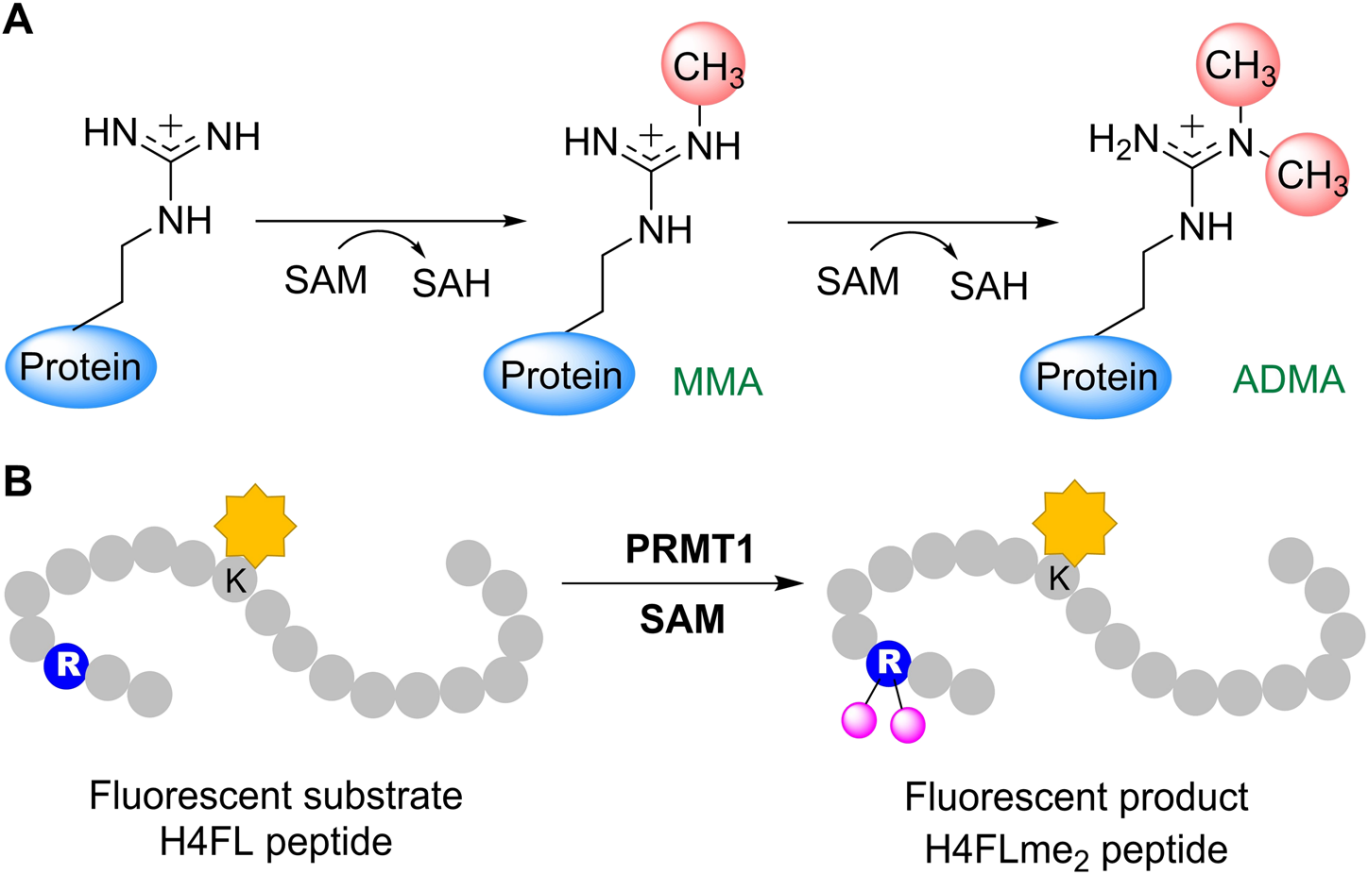
Arginine methylation by PRMT1. **a** PRMT1-mediated methylation reaction. **b** Use of the fluorescent peptide H4FL to study PRMT1-mediated arginine methylation

**Fig. 2:**
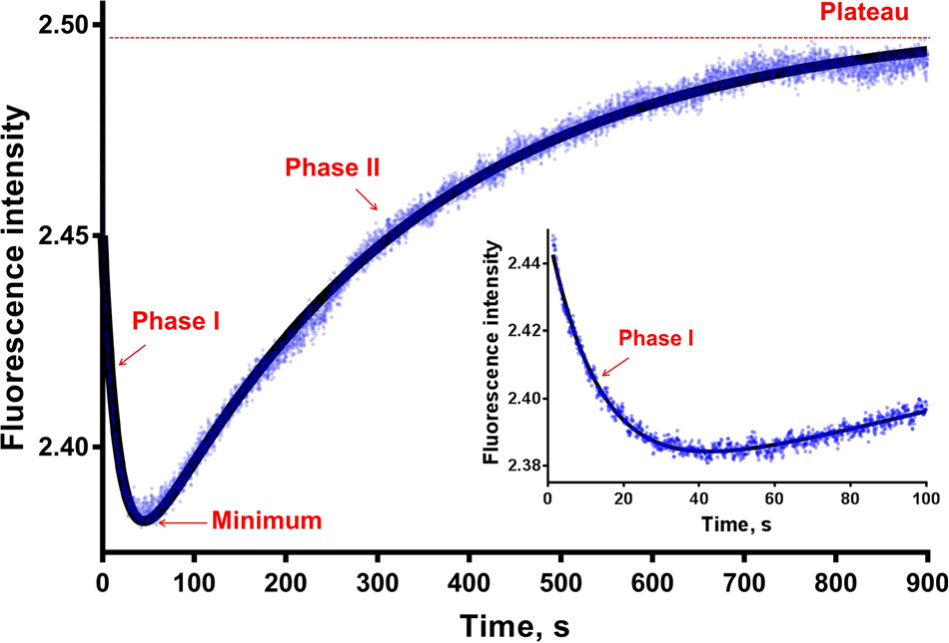
Time course of PRMT1 methylation (1–900 s). The raw data (total of 10,000 points) are shown as blue dots. The curve fitted by equation 2 is shown as a solid black line, which is the average of 4 or 5 replicates. A magnified view of 1–100 s is shown on the side. The concentrations of the enzyme, cofactor and substrate are as follows: [PRMT1] = 0.2 µM, [SAM] = 3.5 µM, [H4FL] = 0.4 µM

PRMT1 is the major type I enzyme responsible for asymmetric arginine dimethylation.^31^ PRMT1 transfers the methyl group from SAM to a guanidine nitrogen of arginine to form MMA, which can be further methylated into ADMA (Fig. 1a).^31^ Stopped flow is a powerful technique to study the transient kinetics of enzymes.^32^ Recently, by detecting the intrinsic tryptophan fluorescence changes of PRMT1, along with global fitting analysis, we elucidated the major kinetic steps of PRMT1 catalysis with resolved rate constants (*k*_on_ and *k*_off_) for individual steps, which provided important mechanistic insights of how PRMTs interact with their substrates and catalyze the methyl transfer reaction.^31^ In addition, we designed and synthesized fluorescein-labeled substrate peptides as fluorescent reporters to probe the arginine methylation reaction.^33,34^ One of these probes is a fluorescein-labeled 20-residue histone H4 N-terminal tail peptide: acetyl-SG**R**GKGGKGK(FL)GKGGAKRHRK (abbreviated as H4FL), in which the methylation site resides on arginine-3 and the fluorescein group is attached to the side chain of residue lysine-10 (Fig. 1b). The kinetic parameters of this fluorescent peptide in PRMT1 catalysis are comparable to the natural substrate,^35^ the 20-residue histone H4 N-terminal tail peptide (H4-20, acetyl-SGRGKGGKGKGKGGAKRHRK): the *K*_m_ and *k*_cat_ values for H4-20 are 0.64 ± 0.04 µM and 0.81 ± 0.01 min^−1^, while the *K*_m_ and *k*_cat_ values for H4FL are 0.50 ± 0.05 µM and 0.43 ± 0.01 min^−1^. In the previous study, we observed a biphasic progression curve for the PRMT1-catalyzed methylation of H4FL under conditions in which the cofactor SAM saturates the enzyme ([PRMT1] = 2 µM, [SAM] = 100 µM, [H4FL] = 0.4 µM).^35^ To further corroborate these results, we measured the stopped flow fluorescence curve of PRMT1 catalysis under a different condition ([PRMT1] = 0.2 µM, [SAM] = 3.5 µM, [H4FL] = 0.4 µM) and the same pattern of progression curves was observed, in which there was a decay phase (Phase I) until a minimum (the lowest point) was reached, followed by an increasing phase (Phase II) until it approached a plateau, as illustrated in Fig. 2.

A critical question is regarding the mechanism underlying the biphasic nature of the H4FL methylation progression curve (i.e., the fluorescence first decreases and then goes up). During the progression of the arginine methylation reaction, the substrate peptide H4FL, mono-methylated peptide H4FLme_1_ (acetyl-SG**Rme**GKGGKGK(FL)GKGGAKRHRK), asymmetric dimethylated peptide H4FLme_2_ (acetyl -SG**Rme**_**2a**_GKGGKGK(FL)GKGGAKRHRK) and many intermediate binary or ternary complexes are involved. Based on our established PRMT1 kinetic model,^31^ we used KinTek Explorer 5.2 to simulate the concentration changes of the relevant species during the methylation time course under the conditions of [PRMT1] = 0.2 µM, [SAM] = 3.5 µM and [H4FL] = 0.4 µM (Figure S1A). At the very beginning of the reaction, substrate H4FL quickly forms an enzyme-H4FL complex (E•H4) and an enzyme-cofactor-H4FL complex (E•SAM•H4), and undergoes a conformational change (F•SAM•H4), which is reflected as a sharp decrease in the total free H4FL concentration. Later, the F•SAM•H4 ternary complex produces the mono-methylated product (H4FLme_1_), and the concentration of free H4FLme_1_ increases while the concentration of free H4FL decreases. H4FLme_1_ can form binary or ternary complexes with the enzyme and the cofactor during this process. The mono-methylated peptide is further converted into the dimethylated product, H4FLme_2_. As the reaction moves forward, the concentrations of substrate H4FL and H4FLme_1_ continue to decrease while the H4FLme_2_ concentration increases. The concentrations of all the other binary or ternary intermediates also decrease, except for those of E•H4me_2_, E•SAM•H4me_2_ and F•SAM•H4me_2_. Indeed, the total concentration of the free peptides ([H4FL] + [H4FLme] + [H4FLme_2_]) follows a biphasic pattern in which they first decrease and then increase (Figure S1C), which results from the dynamics of complex formation and product release during the methylation process. Overall, the expected conversion of the substrate H4FL to the intermediate H4FLme_1_ and to the product H4FLme_2_ is observed.

Determining the relative fluorescence intensity values of the free peptides and the related complexes is the key to deconvolving the biphasic fluorescent signal changes in the progression course. During the binary complex formation of the fluorescein-labeled peptides (H4FL, H4FLme_1_, and H4FLme_2_) with PRMT1, we noticed that the fluorescent signal decreased over time.^35^ The observed overall fluorescence intensity can be defined as f_p_ · [H4FL] + f_c_ · [E•H4FL], in which the fluorescence factor is *f*_p_ for the free peptide and *f*_c_ for the complex, both in units of µM^−1^. In the stopped flow assays, we mixed excess amounts of PRMT1 in three concentrations (2, 4, or 6 µM) with H4FL, H4FLme_1_ and H4FLme_2_ peptides at 0.4 µM. The raw data in Figure S2, Figure S3, and Figure S4 were analyzed by the global fitting function of the KinTek Explorer 5.2 software, based on the reported *k*_on_ and *k*_off_ values of each fluorescent peptide.^35^ For H4FL, the values of *f*_p_ and *f*_c_ are 6.4 and 5.6 µM^−1^; for H4FLme_1_, the values of *f*_p_ and *f*_c_ are 5.4 and 3.8 µM^−1^; and for H4FLme_2_, the values of *f*_p_ and *f*_c_ are 5.9 and 3.9 µM^−1^. Not surprisingly, *f*_p_ is always larger than *f*_c_. The *f*_p_ values of the three fluorescent peptides are similar, possibly because the small size of the methyl group and the long distance between the methylation site, arginine-3, and the fluorescein on lysine-10, which likely minimizes the effect of methylation on the fluorescence. Upon binding with PRMT1, the fluorescein group on the peptide substrate is likely exposed to a different physicochemical environment, which results in reduced fluorescence of the complexes.

Based on these observations and analysis, we propose that the two phases of the H4FL methylation time course are the overall result of the concentration changes of the species involved in the methylation process and their differences in fluorescence intensity. The fluorescence intensity of the free peptides is relatively higher than that of their corresponding ligand-PRMT1 complexes. Consequently, upon mixing the reaction components, H4FL first forms binary or ternary complexes with E and SAM while the amount of free H4FLme_1_ or H4FLme_2_ is very small, which leads to a decreased total concentration of the free peptides (Figure S1C) reflected as a decreasing curve in Phase I (Fig. 2). In the later stage, as more substrates have reacted, the methylated products are formed and released from the enzyme due to its intrinsic low affinity,^17^ and the total concentration of the free peptides increases (Figure S1C), which is reflected as an increasing curve in Phase II (Fig. 2). In the parallel stopped flow fluorescence experiments using H4FL, H4FL_me1_ and H4FL_me2_ peptides under the same conditions ([PRMT1] = 0.2 µM, [SAM] = 3.5 µM, [H4 peptides] = 0.4 µM), we observed a very similar biphasic pattern for the H4FL and H4FL_me1_ methylation curves. However, the H4FL_me2_ curve showed a strong decreasing trend in Phase I upon mixing but a minimal increase in Phase II (Figure S7). These results together suggested that the methylation process of the fluorescein-labeled substrate is represented by the overall fluorescence intensity change, in which Phase I is likely related to peptide substrate binding while Phase II is likely related to peptide product formation.

### Use of the stopped flow fluorescence assay for PRMT1 inhibition measurement

Compared to non-continuous methods, stopped flow technology can rapidly mix two components within a few milliseconds and then measure the fluorescent signals at any stage of the reaction to continuously monitor the entire process. The total progression time curve can provide mechanistic and quantitative information of how an enzyme regulator, such as a small molecule inhibitor, affects the enzymatic reaction. In the stopped flow fluorescence assay, we chose the balanced condition ([PRMT1] = 0.2 μM, [H4FL] = 0.4 μM and [SAM] = 3.5 μM), in which the concentrations of substrate H4FL and cofactor SAM are close to their *K*_m_ values (the *K*_m_ of H4FL is 0.50 ± 0.05 μM, and the *K*_m_ of SAM on H4FL is 3.1 ± 0.46 μM),^35,36^ to indiscriminately characterize competitive, uncompetitive and noncompetitive PRMT1 inhibitors. Again, the methylation time course of the H4FL peptide under this condition exhibited a biphasic pattern: the fluorescence first decreased until a minimum (at 40 seconds), then increased to a plateau (Fig. 2). This biphasic time course was analyzed using a double-exponential equation (equation 2), which resulted in five parameters: *a = *0.06974 ± 1.90E−04, *k*_1_ = 0.06161 ± 3.34E−04 s^−1^, *b* = −0.1115 ± 9.25E−05, *k*_2_ = 0.004804 ± 1.15E−05 s^−1^ and *c* = 2.501 ± 1.03E−04 (Table S1A). The first part of the equation, *F*1 = *a* · *exp*(−*k*_1_ · *t*), is dominant in Phase I (the decay phase), where the amplitude parameter *a* and the rate constant *k*_1_ (s^−1^) together describe the decreasing trend of the curve. The second part of the equation, *F*2 = *a* · *exp*(−*k*_2_ · *t*), is dominant in Phase II (the increasing phase), where the amplitude parameter *b* and rate constant *k*_2_ (s^−1^) together describe the increasing trend of the curve. The obtained value of parameter *c* is the fluorescence intensity at the plateau, which represents the reaction endpoint. The simulation results (Figure S1) showed that the concentration of the product was very close to the plateau at 900 s, which suggested that almost all the substrate had turned over after 900 s under the experimental conditions ([PRMT1] = 0.2 µM, [SAM] = 3.5 µM, [H4FL] = 0.4 µM). The value of *c* can be normalized to any number without affecting the fluorescence amplitude values (*a* and *b*) or the rate constant values (*k*_1_, *k*_2_).

To obtain the IC_50_ of a specific inhibitor, the relative activities of the enzyme in presence of different concentrations of the inhibitor are required (equation 1).^37^ In a typical non-continuous assay (e.g., a radiometric filter binding assay), we need to determine a time course under the desired experimental conditions with an optimal reaction time. The chosen reaction time should stay within the initial conditions to ensure the relationship between the readouts, i.e., counts per minute (CPM) and time (*t*), is linear. Within this period, the concentration of the product has minimal influence on the rate of the reaction, and the reaction time course can be described as *y*=*K* · t, where *K* is the rate of the reaction (e.g., µM s^−1^) as well as the slope of the linear curve. By adding different concentrations of the inhibitor to the reaction mixture, the relative activity of the enzyme can be obtained by normalizing *K* to the reaction rate without the inhibitor, and then calculate the IC_50_ value using equation 1. In our stopped flow fluorescence assay, the reaction rate of Phase I and Phase II can be obtained by approximating and deriving the double-exponential equation (equation 2). In Phase I, during a very short period of time (where *t* is small), the curve is nearly linear, and the exponential equation can be approximately described as F1 = *a* · (−*k*_1_) · *t*. Therefore, the derivation of F1 equals –*k*_1_∙*a*, which is the slope of Phase I. Similarly, the curve of Phase II at early stage can be approximately described as F2 = *b* · (−*k*_2_) · *t*. This derivation of F2 equals –*k*_2_∙*b*, which is the slope of Phase II. The slopes at various inhibitor concentrations, normalized to the slope in the absence of inhibitor, can be used to obtain the corresponding relative enzyme activity from which the potency value (IC_50_) of the inhibitor can be calculated with equation 1.

### Effect of enzyme concentration on the stopped flow time course

**Fig. 3:**
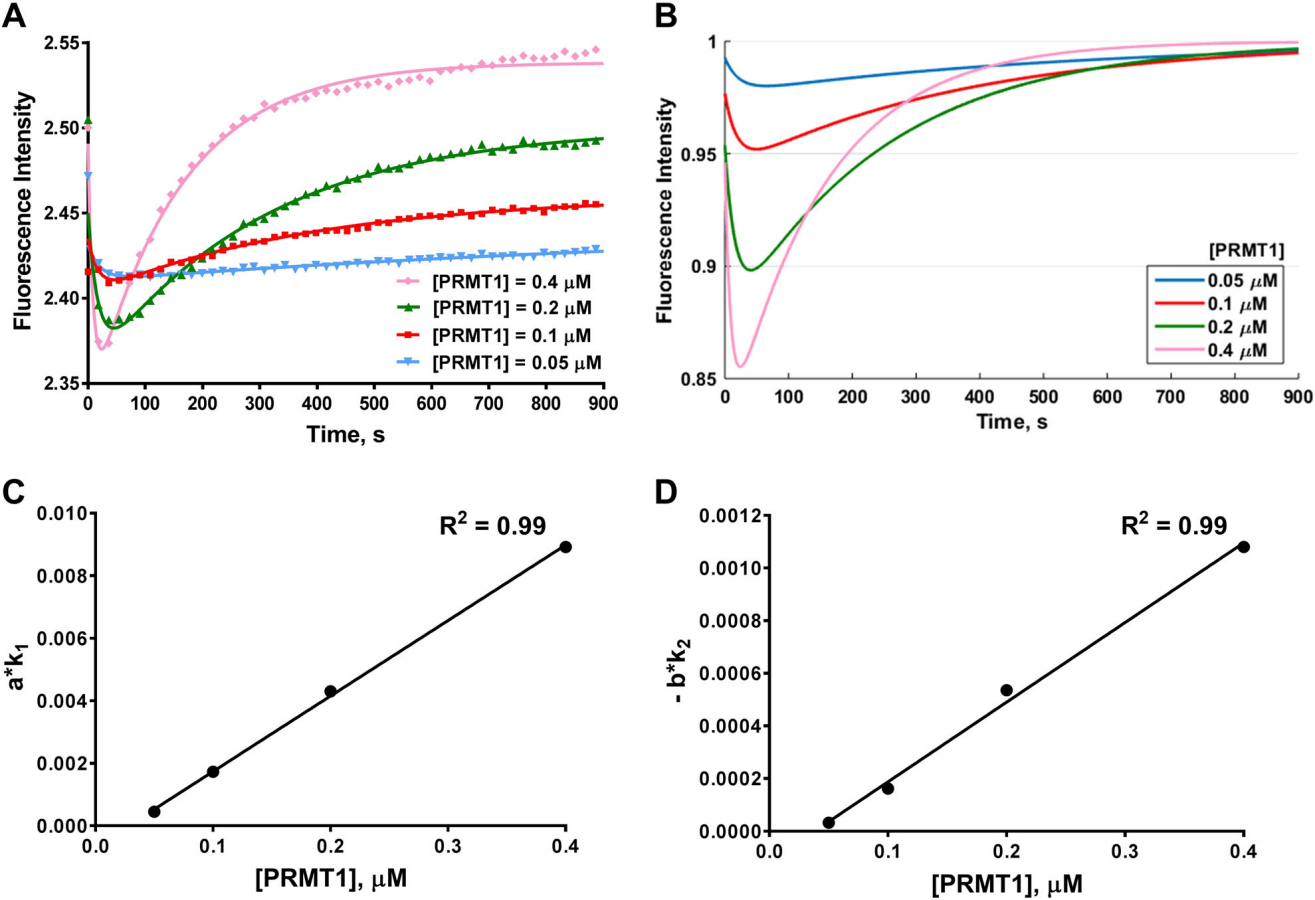
Time course of PRMT1 methylation with varied enzyme concentrations. **a** The curves were fitted with equation 2 to generate the values in Table S1A. Each fitting curve used 10,000 data points, but only 50 data points are shown. Each curve is the average of 4 to 6 replicates. **b** shows the simulation results from the values in Table S1A at fixed c = 1. **c**, **d** represent the relationship of the slope values (*a*·*k*_1_ and *b*·*k*_2_) with varying concentrations of PRMT1 (values listed in Table S1B). The linear fitting curves are shown as solid black lines. The concentrations of the cofactor and the substrate were fixed at [SAM] = 3.5 µM and [H4FL] = 0.4 µM in these experiments

First, to test the effects of enzyme inhibition on the stopped flow fluorescence response, we measured the fluorescence time courses with concentrations of PRMT1 ranging from 0.05 to 0.4 µM. The obtained progression curves are shown in Fig. 3a, b, which were fitted by equation 2. The calculated *a*, *b*, *k*_1_, *k*_2_ values are summarized in Table S1A. Since the concentration of the substrate H4FL was fixed for all the reactions, we can arbitrarily normalize the plateau fluorescence intensity value *c* to 1 (Fig. 3b). When the enzyme concentration was increased, the minima shifted from 65 to 24 s, and the shape of the curve near the minimum became sharper (Fig. 3b), which indicated that the rates of both Phase I and Phase II were increased when more PRMT1 was present in the reaction mixture. Indeed, the values of *a*, *k*_1_, −*b* and *k*_2_ increased with increasing concentration of PRMT1: *a* increased from 0.01079 to 0.09437, *k*_1_ increased from 0.04187 to 0.09452 s^−1^, −*b* increased from 0.01117 to 0.156 and *k*_2_ increased from 0.00287 to 0.006924 (Table S1A). The calculated slope of Phase I increased from 4.52E−04 to 8.92E−03 s^−1^, and the slope of Phase II increased from 4.52E−04 to 8.92E−03 s^−1^ (Table S1B). When we plotted *a · k*_1_ or −*b · k*_2_ values with respect to the PRMT1 concentration, a linear relationship was observed (Fig. 3c,d**)**. This result indicated that the initial rates, reflected by the slope values of Phase I and Phase II, were linearly proportional to the concentration of PRMT1 under the assay conditions.

### Effect of cofactor SAM concentration on the stopped flow time course

**Fig. 4:**
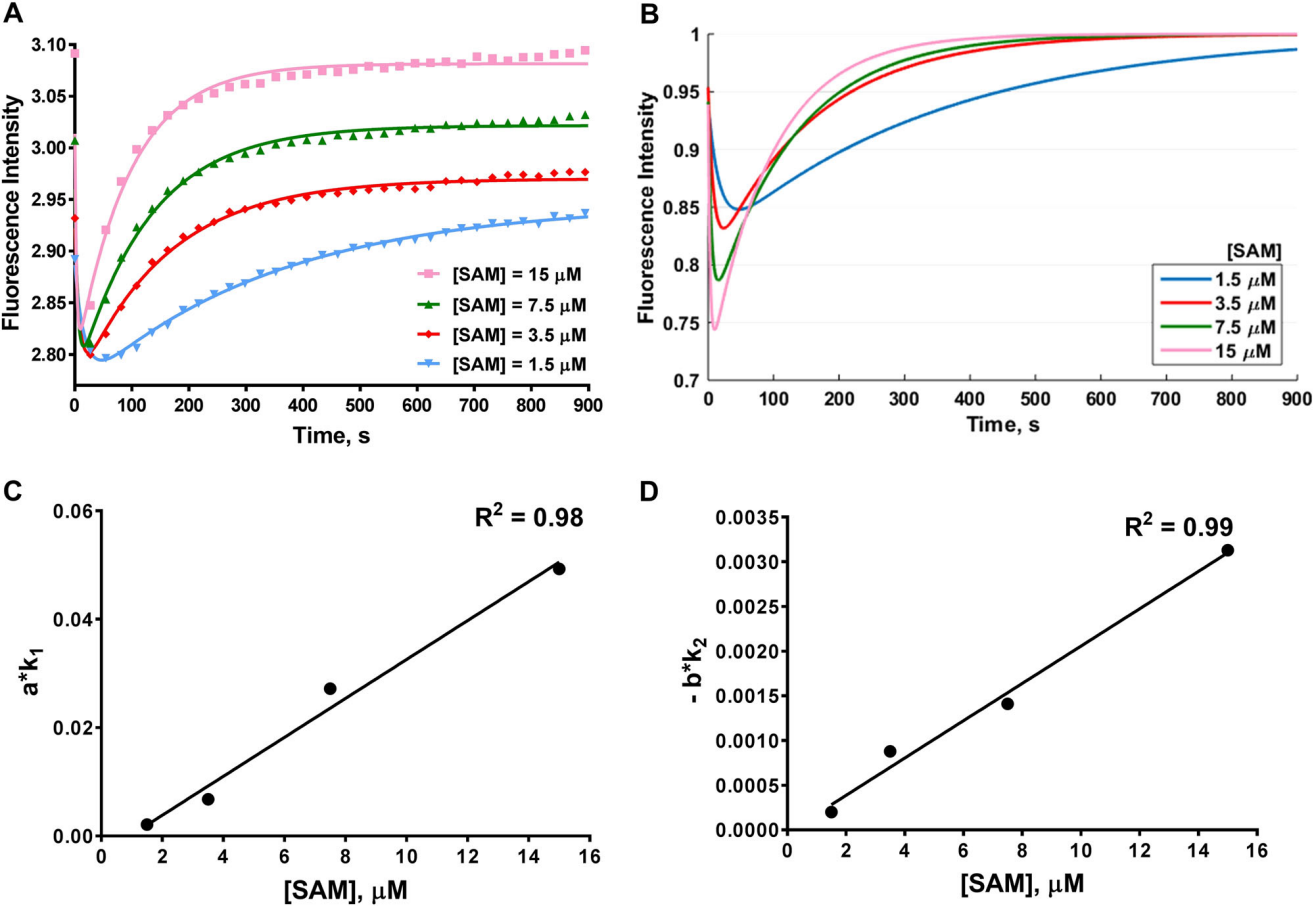
Time course of PRMT1 methylation with varied cofactor concentrations. **a** The curves were fitted with equation 2 to generate the values in Table S2A. Each fitting curve used 10,000 data points, but only 50 data points are shown. Each curve is the average of 4 to 6 replicates. **b** The simulation results from the values in Table S2A at fixed c = 1. **c**, **d** The relationship of slope values (*a*·*k*_1_ and *b*·*k*_2_) with varying concentrations of SAM (values listed in Table S2B). The linear fitting curves are shown as solid black lines. The concentrations of the enzyme and the substrate were fixed at [PRMT1] = 0.4 µM and [H4FL] = 0.4 µM in these experiments

After we measured the effects of enzyme concentration on the stopped flow response, we measured stopped flow fluorescence at various SAM concentrations (1.5 µM, 3.5 µM, 7.5 µM and 15 µM). The obtained progression curves are shown in Fig. 4a, b, and the calculated *a*, *b*, *k*_1_, *k*_2_ values are summarized in Table S2A. For clarity, we again normalized the plateau fluorescence intensity value *c* to 1 (Fig. 4b). The time at which the minimum was reached decreased as the SAM concentration was increased (i.e., the minima shifted to the left). It was also clear that as the concentration of SAM was increased, the slopes of the curve near the minimum became sharper. The values of all the parameters, *a*, *k*_1_, −*b*, and *k*_2_, increased with increasing concentration of SAM: *a* increased from 0.0482 to 0.1992, *k*_1_ increased from 0.04402 to 0.2476 s^−1^, −*b* increased from 0.07363 to 0.2664 and *k*_2_ increased from 0.002713 to 0.01175 (Table S2A). We plotted the values of *a · k*_1_ or −*b · k*_2_ against the SAM concentration (Fig. 3c, d). The slope of Phase I increased from 2.12E−03 to 4.93E−02 s^−1^ and the slope of Phase II increased from 2.00E−04 to 3.13E−03 s^−1^ (Table S2B). The above results indicated that the slopes of Phase I and Phase II are linearly related to the concentration of SAM.

### Detection of PRMT1 inhibition by SAM-competitive inhibitors SAH and Sinefungin

**Fig. 5:**
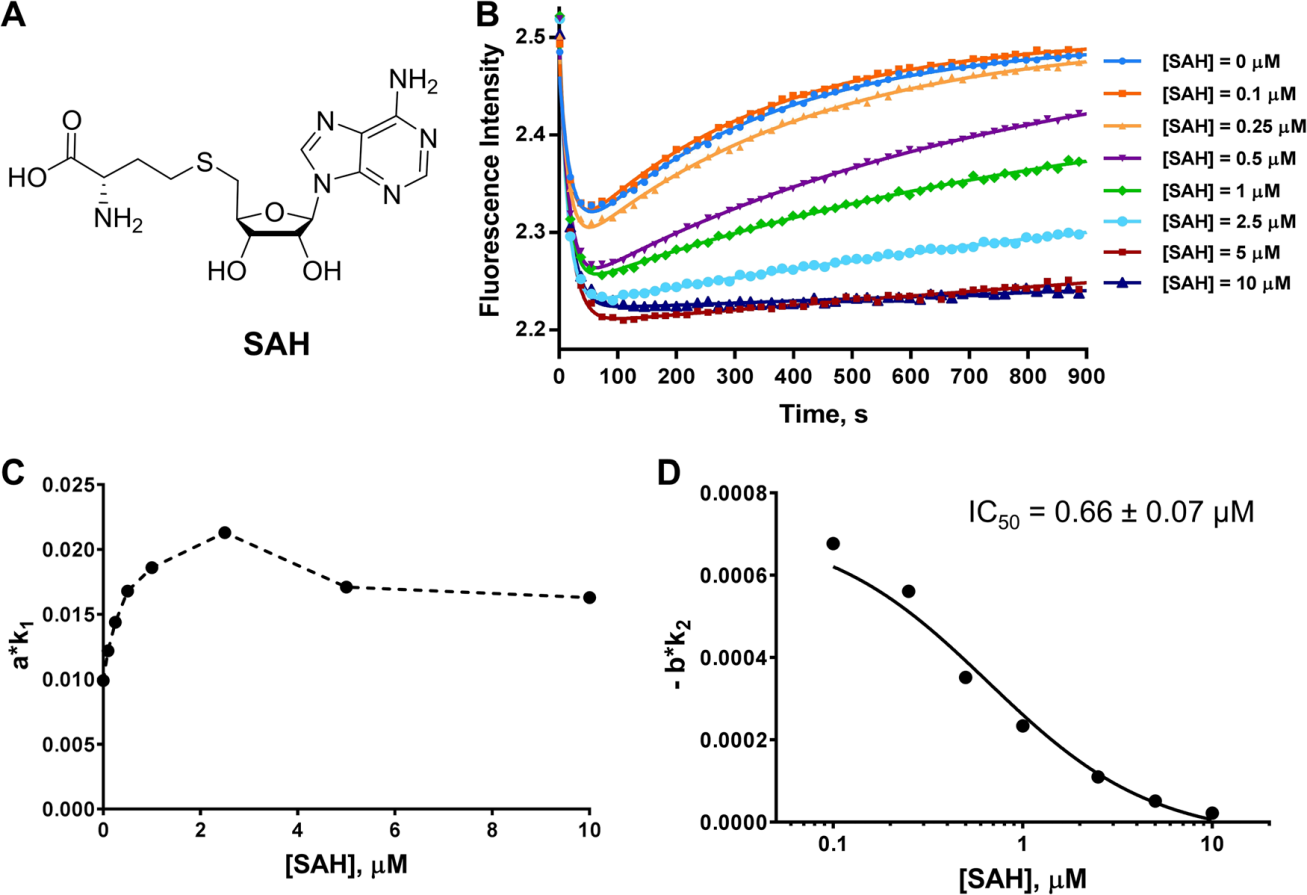
Stopped flow fluorescence assay of the cofactor-competitive inhibitor SAH. **a** Structure of SAH. In **b**, the curves were fitted with equation 2 to generate the values in Table S3A. Each curve used 10,000 data points, but only 50 data points are shown. Each curve is the average of 4 to 6 replicates. **c**, **d** represent the relationships of *a*·*k*_1_ and *b*·*k*_2_ with inhibitor concentrations (values listed in Table S3B). In D, the IC_50_ was calculated using equation 1. The reaction conditions used for all the experiments were [PRMT1] = 0.2 µM, [SAM] = 3.5 µM, and [H4FL] = 0.4 µM, with varying concentrations of SAH

With the assay conditions defined ([PRMT1] = 0.2 μM, [H4FL] = 0.4 μM and [SAM] = 3.5 μM), we examined the changes of the stopped flow fluorescence time course in response to different PRMT1 inhibitors. First, we tested the inhibition of PRMT1 by the SAM analog, SAH (Fig. 5a). With a series of concentrations of SAH added to the mixture, the obtained progression curves clearly showed that the reaction was inhibited by SAH in a dose-dependent manner (Fig. 5b). When the concentration of SAH was 0.1 µM, there was very little difference compared to the control experiment without SAH. When the concentration of SAH was increased to 10 µM, the increasing trend of Phase II was almost abolished. Higher concentrations of SAH resulted in steeper decreasing curves in Phase I and milder increasing curves in Phase II (Fig. 5b). The changes of the parameters, *a*, *k*_1_, −*b*, and *k*_2_ did not follow a simple rule based on SAH concentrations (Table S3A). The relationship between the slopes *a*∙*k*_1_ and −*b*∙*k*_2_ with respect to the SAH concentration is shown in Fig. 5c, d, and the corresponding values are listed in Table S3B. Interestingly, the *a*∙*k*_1_ values of Phase I were elevated when the inhibitor concentration was lower than that of SAM (from 0.1 to 2.5 μM); and when the inhibitor concentration was higher than that of SAM, the *a*∙*k*_1_ values started to decrease (Fig. 5c and Table S3B). The initial slopes of Phase II, −*b*∙*k*_2_, decreased as more SAH was added to the reaction mixture (Fig. 5d), which indicated a dose-dependent inhibitory effect. We used the dose response of −*b*∙*k*_2_ to determine that the IC_50_ of SAH was 0.66 ± 0.07 µM, which falls into the range of the IC_50_ value reported in the literature.^9^

We performed a similar stopped flow experiment for the PRMT1 inhibitor sinefungin (Figure S5), another SAM analog and a universal methyltransferase inhibitor.^38,39^ The obtained reaction curves showed that sinefungin inhibited PRMT1 activity in a dose-dependent manner (Figure S5), very similar to SAH inhibition (Fig. 5). The relationships between *a∙k*_1_ and −*b∙k*_2_ with the sinefungin concentration are shown in Figure S5C and S5D. The values of all parameters are listed in Table S4. Like SAH inhibition, the *a∙k*_1_ values of Phase I increased with higher concentrations of sinefungin up to 3 µM, followed by a slight decrease (Figure S5C). The value −*b∙k*_2_ decreased when more inhibitor was present, and the IC_50_ of sinefungin was calculated to be 0.12 ± 0.08 µM, close to previously reported data.^7,9,10^ These results indicate that the parameter −*b∙k*_2_ of Phase II is appropriate for quantitative characterization of the potency of SAM-competitive inhibitors.

### Detection of PRMT1 inhibition by substrate-competitive inhibitor H4R3me2a

**Fig. 6:**
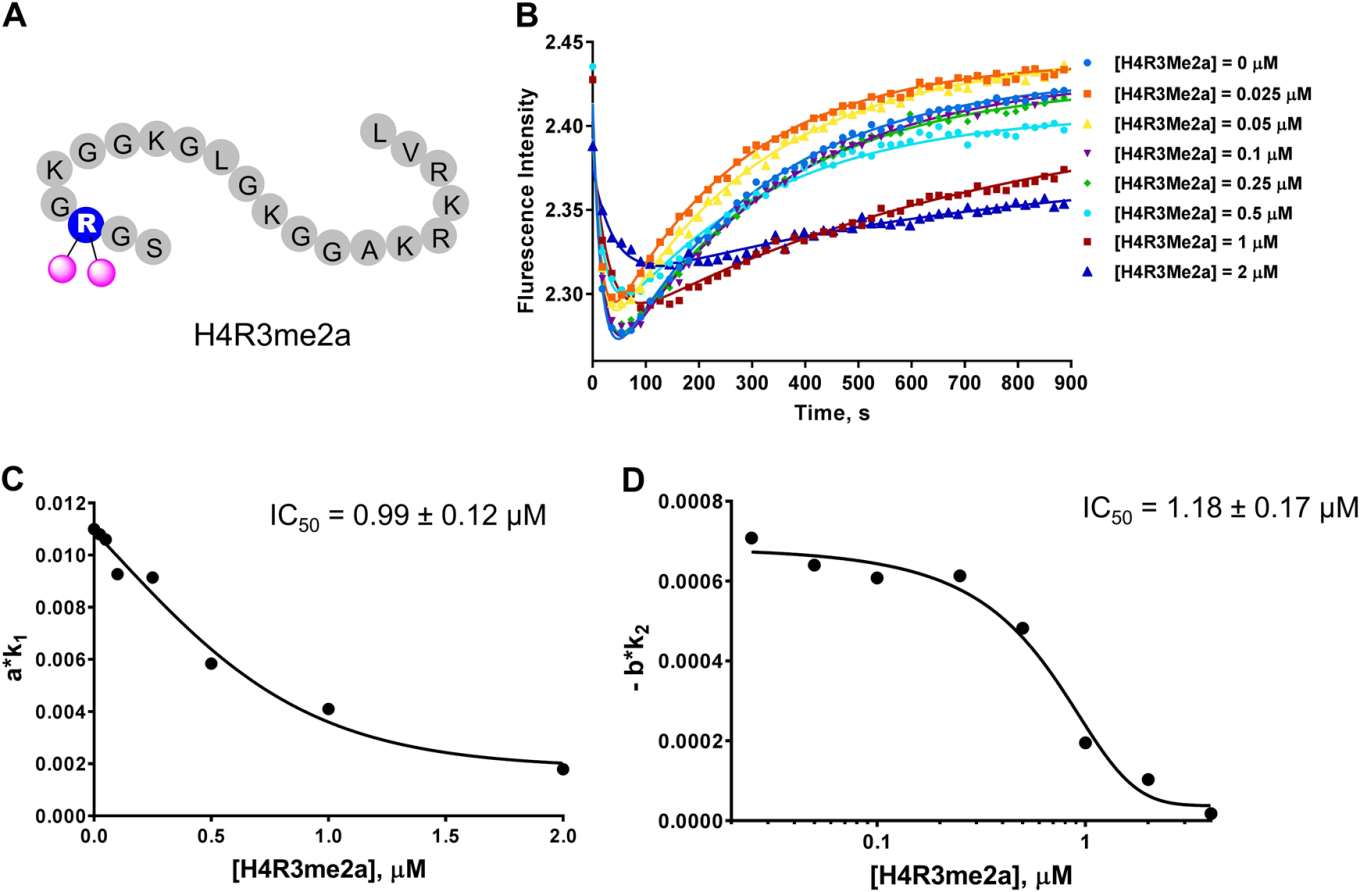
Stopped flow fluorescence assay of the substrate-competitive inhibitor H4R3Me2a. **a** Illustration of H4R3Me2a. In **b**, the curves were fitted with equation 2 to generate the values in Table S5A. Each curve used 10,000 data points, but only 50 data points are shown. Each curve is the average of 4 to 6 replicates. **c** and **d** represent the relationships of a·*k*_1_ and b·*k*_2_ with inhibitor concentrations (values listed in Table S5B). In **c** and **d**, the IC_50_ was calculated using equation 1. The reaction conditions used for all experiments were [PRMT1] = 0.2 µM, [SAM] = 3.5 µM, and [H4FL] = 0.4 µM, with varying concentrations of H4R3Me2a

Next, we investigated the stopped flow response to the product inhibitor, i.e., the asymmetrically dimethylated H4 peptide H4R3me2a (acetyl-SGRme_2a_GKGGKGLGKGGAKRHRKVL) (Fig. 6a). The obtained stopped flow fluorescence curves of the H4R3me2a inhibition assay are shown in Fig. 6b. When the concentration of H4R3me2a was as low as 0.25 µM, no obvious difference was observed (Fig. 6b). When the H4R3me2a concentration was above 0.5 μΜ, the shape of the curves changed significantly, with shallower minima and milder slopes in Phase II. Again, the changes of *a*, *k*_1_, −*b* and *k*_2_ did not follow a simple rule with respect to the inhibitor concentration (Table S5A). However, the slopes *a · k*_1_ and −*b∙k*_2_ clearly showed a dose-dependent inhibition pattern. Unlike in the SAH or sinefungin assays, Phase I was strongly inhibited by increasing concentrations of H4R3me2a (Fig. 6c), and the *a · k*_1_ values decreased from 1.10E−02 ± 5.14E−05 s^−1^ to 1.79E−03 ± 1.72E−05 s^−1^ (Table S5B). Phase II showed a similar pattern as the SAM-competitive inhibition: −*b∙k*_2_ values were reduced when more inhibitors were present (Fig. 6d and Table S5B). The IC_50_ of H4R3me2a calculated from the *a∙k*_1_ curve (Fig. 6c) was 0.99 ± 0.12 µM, and that from the −*b∙k*_2_ curve was 1.18 ± 0.17 µM (Fig. 6d). The two IC_50_ values are very comparable, and both are close to the IC_50_ determined by a radiometric filter binding biochemical assay, which was 1.32 ± 0.20 µM (Figure S6).

### Detection of PRMT1 inhibition by small molecule inhibitor DB75

**Fig. 7:**
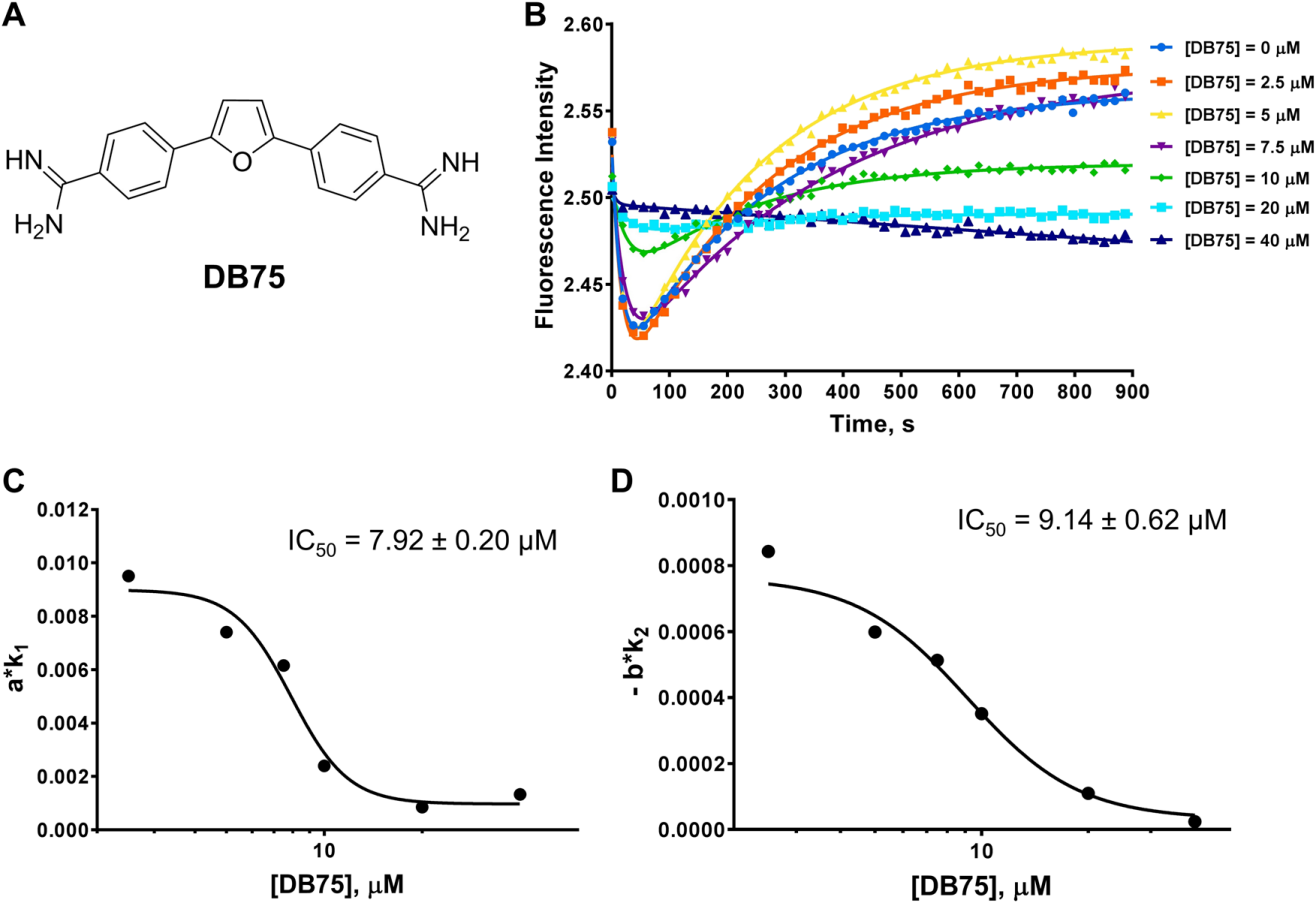
Stopped flow fluorescence assay of DB75. **a** Structure of DB75. In **b**, the curves were fitted with equation 2 to generate the values in Table S6A. Each curve used 10,000 data points, but only 50 data points are shown. Each curve is the average of 4 to 6 replicates. In **c** and **d**, the IC_50_ was calculated using equation 1. The reaction conditions used for all experiments were [PRMT1] = 0.2 µM, [SAM] = 3.5 µM, and [H4FL] = 0.4 µM, with varying concentrations of DB75

Next, we performed stopped flow characterization on recently reported small molecule inhibitors of PRMT1. DB75 (furamidine) is a diamidine molecule with a rigid, crescent-shaped planar scaffold (Fig. 7a). According to our previous study,^40^ its IC_50_ for PRMT1 is 9.4 ± 1.1 µM and it shows a favorable inhibition selectivity against PRMT1 compared to other PRMT members: 42-fold over CARM1, 30-fold over PRMT6 and more than 15-fold over PRMT5. We tested DB75 using the stopped flow fluorescence assay and obtained methylation curves for a series of DB75 concentrations (Fig. 7b). As the inhibitor concentration was increased, the minima of the curves leveled up and the Phase I and Phase II slopes became less steep. When the concentrations of DB75 were above 10 µM, Phase II was almost fully inhibited. The relationships between *a∙k*_1_ and −*b∙k*_2_ with the inhibitor concentration are plotted in Fig. 7c,d, with their corresponding values listed in Table S6B. From this measurement, we observed that both *a∙k*_1_ of the first phase and −*b∙k*_2_ of the second phase were strongly inhibited, in a pattern similar to that of H4R3me2a inhibition, suggesting that DB75 is a substrate-competitive inhibitor. This conclusion is in good agreement with our previous steady-state kinetic analysis that DB75 is primarily competitive with the substrate.^40^ From the *a∙k*_1_ curve, the IC_50_ of DB75 was calculated to be 7.9 ± 0.2 µM; from the −*b∙k*_2_ curve, the IC_50_ was 9.1 ± 0.6 µM, highly consistent with the result from the radiometric filter binding assay, which gave a value of 9.4 ± 1.1 µM.^40^

### Detection of PRMT1 inhibition by MS023

**Fig. 8:**
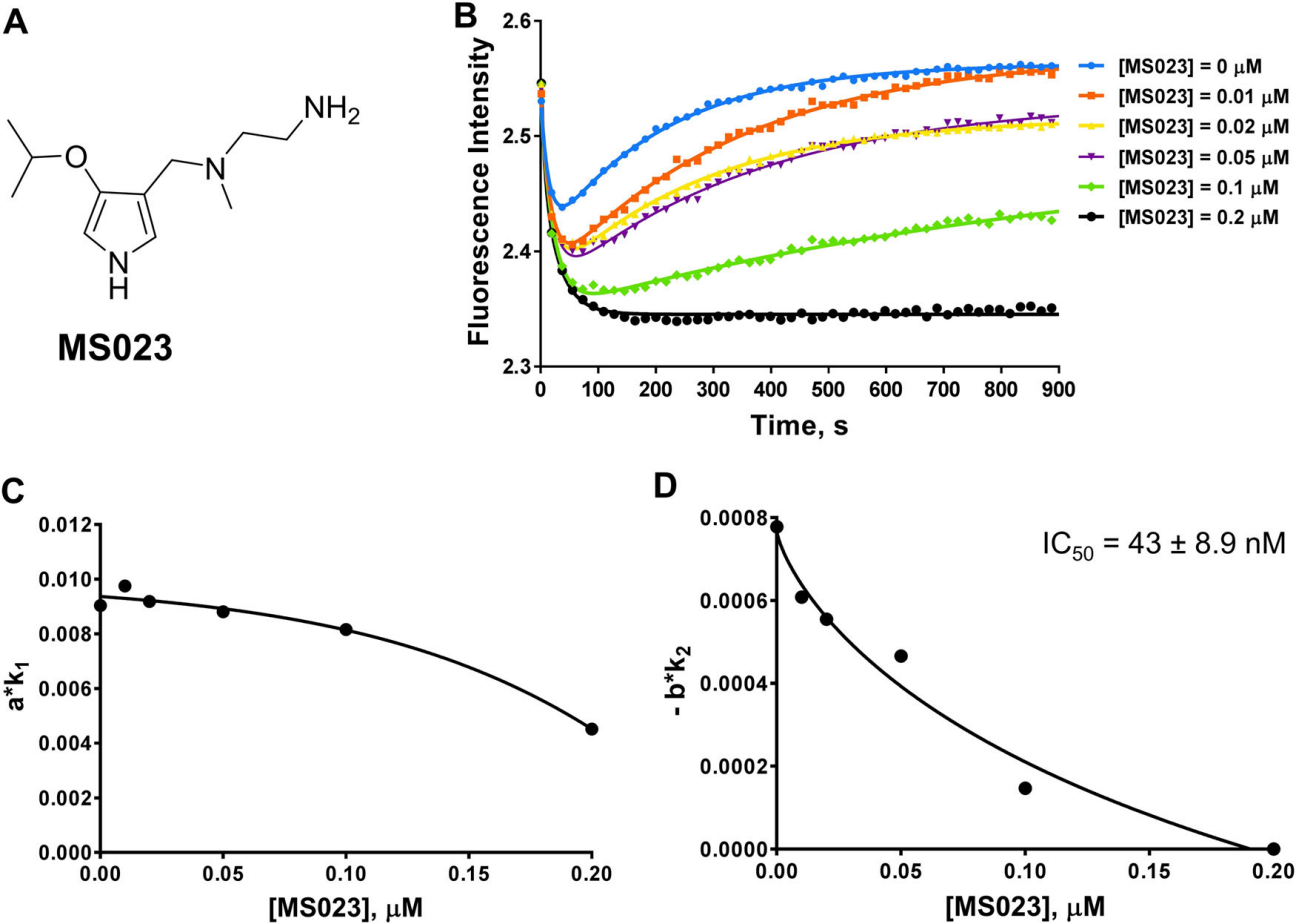
Stopped flow fluorescence assay of MS023. **a** Structure of MS023. In **b**, the curves were fitted with equation 2 to generate the values in Table S7A. Each curve used 10,000 data points, but only 50 data points are shown. Each curve is the average of 4 to 6 replicates. In **c** and **d**, the IC_50_ was calculated using equation 1. The reaction conditions used for all experiments were [PRMT1] = 0.2 µM, [SAM] = 3.5 µM, and [H4FL] = 0.4 µM, with varying concentrations of MS023

Lastly, we performed the stopped flow florescence assay with another small molecule inhibitor, MS023 (Fig. 8a), a type I PRMT inhibitor recently discovered by Kaniskan et al. with an IC_50_ of 30 nM against PRMT1.^41^ In our stopped flow experiment, MS023 was titrated from 10 nM to 200 nM in the reaction mixture. As shown in Fig. 8b, we observed that the higher the concentration of MS023, the milder the slope of Phase II. With 200 nM of MS023, Phase II was almost fully inhibited. The relationships of *a∙k*_1_ and −*b∙k*_2_ with the inhibitor concentration are shown in Fig. 8c, d, and the values are listed in Table S7B. For Phase II, the −*b∙k*_2_ values decreased as more inhibitor was added, and the calculated IC_50_ of MS023 was 43 ± 8.9 nM (Fig. 8d), close to what was previously reported (IC_50_ = 30 nM).^41^ Interestingly, the *a∙k*_1_ values of Phase I modestly decreased with increasing MS023 concentration, and the calculated IC_50_ from this curve was more than 200 nM (Fig. 8c and Table S7B). This result is different from those of substrate-competitive inhibitors (e.g., DB75 and H4R3me2a) or the cofactor-competitive inhibitors (e.g., SAH and sinefungin). The partial inhibition of Phase I by MS023 (Fig. 8c) suggests that MS023 might be a mixed-type noncompetitive inhibitor that is partially substrate-competitive. Indeed, the mechanism of action of MS023 was previously reported to be noncompetitive with both the cofactor SAM and the substrate peptide;^41^ and according to the X-ray co-crystal structure of PRMT6 in complex with MS023, the inhibitor occupied the substrate arginine-binding site.^41^

## Conclusion

There is a strong need to identify PRMT inhibitors for use as novel therapeutic agents to treat diseases and to develop mechanistic tools to investigate the biological functions of PRMTs. The development of PRMT inhibitors relies on robust biochemical assays to evaluate candidate inhibitors. In this work, we have designed a stopped flow fluorescence platform to simultaneously quantitate the potency and characterize the mechanisms of PRMT1 inhibitors. All the observed stopped flow fluorescence progression curves from this transient kinetic assay exhibited a down-and-up biphasic behavior, which suggests the complexity of intermediate species formation throughout the PRMT1-catalyzed methylation process. All the PRMT1 inhibitors blocked Phase II in a dose-dependent manner, from which the initial rate values (*-b · k*_1_) could be used to accurately determine the IC_50_. In contrast, different types of PRMT1 inhibitors showed varying effects on Phase I of the stopped flow curve: SAM-competitive inhibitors affected Phase I in a complex multiphasic manner (Fig. 5c), but substrate-competitive inhibitors showed simple Langmuir isotherm inhibition (Fig. 6c). Overall, the stopped flow fluorescence assay is effective for characterizing the potency of PRMT1 inhibitors and for providing mechanistic insights for MOA investigation. This approach bears the advantages of being homogeneous, nonradioactive, and mix-and-measure in nature, and it allows for continuous measurement of methylation inhibition. We envision that this assay format can be potentially expanded to detect and characterize inhibitors of other histone-modifying enzymes.

## Materials and methods

### Protein expression

Recombinant His-tagged rat PRMT1 was expressed in *E. coli* and purified with Ni-charged His6x-tag binding resin as reported previously.^35,31^ In brief, the N-terminal His-tagged human recombinant PRMT1 (PRMT1 residues 11–353, UniProt entry Q99873) was cloned into the pET28b^(+)^ vector and transformed into BL21(DE3) cells (Stratagene, CA, USA) by heat shock. Transformed bacteria were incubated in LB media at 37 °C for growth and then at 16 °C for protein expression with 0.3 mM IPTG induction. Cells were harvested by centrifugation and lysed by a microfluidics cell disrupter. The supernatant containing the PRMT1 protein was loaded onto Ni-charged His6x-tag binding resin (Novagen, WI, USA) in equilibrium buffer (25 mM Na-HEPES, pH 7.0; 300 mM NaCl; 1 mM PMSF; and 30 mM imidazole). Beads were washed thoroughly with washing buffer (25 mM Na-HEPES, pH 7.0; 300 mM NaCl; 1 mM PMSF; and 70 mM imidazole), and protein was eluted with elution buffer (25 mM Na-HEPES, pH 7.0; 300 mM NaCl; 1 mM PMSF; 100 mM EDTA; and 200 mM imidazole). Protein purity was checked by 12% SDS-PAGE, and concentrations were determined by the Bradford assay.^42^

### Peptide synthesis

In all the stopped flow fluorescence assays, H4FL peptides (the N-terminal 20 amino acids of histone H4, with Leu-10 replaced by fluorescein-labeled Lys-10) were used as probes.^33^ H4FL was synthesized using the Fmoc-based solid phase peptide synthesis (SPPS) protocol on a PS3 peptide synthesizer (Protein Technology, Arizona, USA) as described previously.^33^ Each amino acid was coupled to the solid phase with HCTU [*O*-(1H-6-chlorobenzotriazole-1-yl)-1,1,3,3- tetramethyluronium hexafluorophosphate] (Novabiochem, Darmstadt, Germany), using 4 equivalents of amino acid. The Fmoc group was deprotected with 20% v/v piperidine/DMF, and the N-terminal amino acid was acetylated with acetic anhydride. The peptide was cleaved from the Wang resin by a cleavage solution consisting of 95% trifluoroacetic acid (TFA), 2.5% H_2_O, and 2.5% triisopropylsilane. It was then precipitated in cold ether and pelleted by centrifugation. Crude peptides were collected and purified using a Varian Prostar instrument equipped with a C18 reversed-phase high-performance liquid chromatography (RP-HPLC) column, where 0.05% TFA in water and 0.05% TFA in acetonitrile were the two mobile phases used for gradient purification. The identity of peptides was confirmed by MALDI-MS. The concentrations of the peptides were calibrated according to the absorption of fluorescein at 492 nm.

### Stopped flow fluorescence assay

In a stopped flow fluorescence assay, the binding of H4FL to PRMT1 (or the PRMT1—cofactor complex) quenches the peptide fluorescence, while release of the peptide restores the fluorescence. The fluorescence signal change was detected at room temperature on an Applied Photophysics Ltd (UK) stopped flow system, using an excitation wavelength of 495 nm and a long pass emission filter centered at 510 nm. The widths of the entrance and exit slits of the monochromator were set to 0.5 mm. An equal volume of samples from two syringes was driven into the observation cell for mixing measurements. The H4FL concentration in all experiments was 0.4 μM. Typically, the enzyme PRMT1 was pre-mixed with H4FL and loaded into one syringe, while the mixture of SAM and H4FL, with or without the inhibitor, was loaded into the other syringe. For inhibition assays, the enzyme and H4FL solution were mixed with the SAM, H4FL and inhibitor solution at the following final concentrations: 0.2 μM PRMT1, 0.4 μM H4FL, 3.5 μM SAM, and a range of concentrations of the various inhibitors. The fluorescent signal was recorded for 900 seconds, with 10,000 data points in total. Data from four to six drives were collected and averaged for each curve.

After averaging the shot data, the association time courses were fitted to a double-exponential function (equation 2) using GraphPad Prism (CA, USA). The methylation time course exhibited two distinct kinetic phases. F is the fluorescence intensity at time *t*, *k*_1_, and *k*_2_ are the rate constants for Phase I and Phase II, *a* is the amplitude of the fluorescence change for *k*_1_, and *b* is the amplitude of the fluorescence change for *k*_2_. Simulation curves based on the values of *a*, *b*, *k*_1_, and *k*_2_ at fixed *c* = 1 were produced using Matlab. The IC_50_ value of inhibitors is determined by equation 1 using GraphPad Prism (CA, USA). Equations 1 and 2 are shown below:

1 $${\mathrm{Relative}}\,{\mathrm{activity = 1/(1 + ([Inhibitor]/IC}}_{{\mathrm{50}}}{\mathrm{))}}$$

2 $${\mathrm{F}} = a \cdot exp\left( { - k_1 \cdot {\mathrm{t}}} \right) + b \cdot \exp \left( { - k_2 \cdot {\mathrm{t}}} \right) + c$$

### Radiometric filter-binding assay for IC_50_ determination of the PRMT inhibitors

Peptide substrate, inhibitor and [^3^H]-SAM were preincubated in the reaction buffer for 2 min prior to the methyl transfer reaction, which was initiated by adding the enzyme (30 µL total volume) at room temperature. The final concentrations of PRMT1, ^3^H-SAM, and H4 peptide were 0.02, 0.5, and 1 μM, respectively. The reaction buffer contained 50 mM HEPES (pH 8.0), 50 mM NaCl, 1 mM EDTA, and 0.5 mM DTT. The reaction was incubated for 10 min and then was quenched with 30 µL of isopropanol, followed by spotting the reaction mixture on separate squares of P81 Ion Exchange Cellulose Chromatography Paper (Reaction Biology Corp, item number: IEP-01). Then, the paper squares were air dried for 30 min before being washed three times with 50 mM NaHCO_3_ (pH 9). After the washed paper squares were dried in air overnight, they were transferred into 3.5 mL vials full of scintillation oil, and the amount of methylation was quantified by scanning the vials with a scintillation counter (Beckman Coulter, California, USA). The background control contained only [^3^H]-SAM and the substrate. The reaction sample readouts, after subtracting the background, were normalized by the reaction without inhibitor and fitted by equation 1 to obtain IC_50_ values. The reported data were based on the average of two experiments.

## Electronic supplementary material

Supporting information
