# Supplementary material for: Detection of PRMT1 inhibitors with stopped flow fluorescence
Source: Signal Transduct Target Ther. 2018 Mar 9;3:6. doi: 10.1038/s41392-018-0009-6 (PMC5843908; doi:10.1038/s41392-018-0009-6)
Supplement: Supplementary file 1 — Supporting information [file 41392_2018_9_MOESM1_ESM.docx]

Detection of PRMT1 Inhibitors with Stopped Flow Fluorescence

Kun Qian, Hao Hu†, Hui Xu, Y George Zheng*

Department of Pharmaceutical and Biomedical Sciences, University of Georgia, Athens, Georgia, 30602, United States.

†Present Address： The Chemical Proteomics Center and State Key Laboratory of Drug Research, Shanghai Institute of Materia Medica, Chinese Academy of Sciences, Shanghai 201203, P.R. China

# Table S1A. Parameter values of the enzyme concentration-dependent time courses*^a^*.

| [PRMT1], µM | a | k_1_, (s^-1^) | b | k_2_, (s^-1^) | R^2^ |
| --- | --- | --- | --- | --- | --- |
| 0.05 | 0.01079 ± 1.32E-04 | 0.04187 ± 1.05E-03 | -0.01117 ± 8.97E-05 | 0.00287 ± 1.03E-04 | 0.9972 |
| 0.1 | 0.03605 ± 1.42E-04 | 0.04810 ± 3.86E-04 | -0.05688 ± 9.80E-05 | 0.002841 ± 1.99E-05 | 0.9898 |
| 0.2 | 0.06974 ± 1.90E-04 | 0.06161 ± 3.34E-04 | -0.1115 ± 9.25E-05 | 0.004804 ± 1.15E-05 | 0.7915 |
| 0.4 | 0.09437 ± 2.85E-04 | 0.09452 ± 5.45E-04 | -0.156 ± 1.35E-04 | 0.006924 ± 1.12E-05 | 0.9977 |

*^a^*The standard deviation values of fitting with equation 2 are shown.

# Table S1B. Slope values*^a^* of the enzyme concentration-dependent time courses*^a^*.

| [PRMT1], µM | a·k_1_, (s^-1^) | -b·k_2_, (s^-1^) |
| --- | --- | --- |
| 0.05 | 4.52E-04 ± 1.26E-05 | 3.21E-05 ± 1.17E-06 |
| 0.1 | 1.73E-03 ± 1.55E-05 | 1.62E-04 ± 1.16E-06 |
| 0.2 | 4.30E-03 ± 2.61E-05 | 5.36E-04 ± 1.35E-06 |
| /0.4 | 8.92E-03 ± 5.81E-05 | 1.08E-03 ± 1.99E-05 |

*^a^*The standard deviation of the slope values are calculated from the fitting standard deviation values of a, b, k_1_ and k_2_.

# Table S2A. Parameter values*^a^* of the cofactor SAM-dependent time courses.

| [SAM], µM | a | k_1_, (s^-1^) | b | k_2_, (s^-1^) | R^2^ |
| --- | --- | --- | --- | --- | --- |
| 1.5 | 0.0482 ± 2.31E-04 | 0.04402 ± 4.06E-04 | -0.07363 ± 8.97E-05 | 0.002713 ± 1.28E-05 | 0.9905 |
| 3.5 | 0.0846 ± 3.83E-04 | 0.08016 ± 6.88E-04 | -0.1161 ± 2.14E-04 | 0.007565 ± 1.73E-05 | 0.9937 |
| 7.5 | 0.1529 ± 1.79E-03 | 0.1778 ± 2.91E-03 | -0.2054 ± 3.40E-04 | 0.006848 ± 1.85E-05 | 0.9883 |
| 15 | 0.1992 ± 2.65E-03 | 0.2476 ± 4.40E-03 | -0.2664 ± 5.21E-04 | 0.01175 ± 3.03E-05 | 0.9886 |

*^a^*The standard deviation values of fitting with equation 2 are shown.

# Table S2B. Slope values*^a^* of the cofactor SAM-dependent time courses.

| [SAM], µM | a·k_1_, (s^-1^) | -b·k_2_, (s^-1^) |
| --- | --- | --- |
| 1.5 | 2.12E-03 ± 2.20E-05 | 2.00E-04 ± 9.72E-07 |
| 3.5 | 6.78E-03 ± 6.58E-05 | 8.78E-04 ± 2.58E-06 |
| 7.5 | 2.72E-02 ± 5.47E-04 | 1.41E-03 ± 4.46E-06 |
| 15 | 4.93E-02 ± 1.09E-03 | 3.13E-03 ± 1.01E-05 |

*^a^*The standard deviation of the slope values are calculated from the fitting standard deviation values of a, b, k_1_ and k_2_.

# Table S3A. Parameter values*^a^* of the SAH inhibition curves.

| [SAH], µM | a | k_1_, (s^-1^) | b | k_2_, (s^-1^) | R^2^ |
| --- | --- | --- | --- | --- | --- |
| 0 | 0.1944 ± 4.03E-04 | 0.05093 ± 1.97E-04 | -0.2213 ± 1.47E-04 | 0.003031 ± 6.73E-06 | 0.9972 |
| 0.1 | 0.2027 ± 4.00E-04 | 0.05995 ± 2.11E-04 | -0.2201 ± 1.26E-04 | 0.003078 ± 5.86E-06 | 0.9978 |
| 0.25 | 0.2214 ± 4.39E-04 | 0.06487 ± 2.18E-04 | -0.2306 ± 1.33E-04 | 0.002434 ± 5.47E-06 | 0.9977 |
| 0.5 | 0.2658 ± 3.49E-04 | 0.06324 ± 1.36E-04 | -0.2629 ± 3.89E-04 | 0.00134 ± 4.32E-06 | 0.9983 |
| 1 | 0.2726 ± 3.65E-04 | 0.06815 ± 1.45E-04 | -0.2134 ± 6.03E-04 | 0.001095 ± 5.70E-06 | 0.997 |
| 2.5 | 0.2961 ± 3.73E-04 | 0.07177 ± 1.40E-04 | -0.1862 ± 2.16E-03 | 0.0005933 ± 9.36E-06 | 0.9944 |
| 5 | 0.2962 ± 3.74E-04 | 0.05757 ± 1.17E-04 | -0.3000 ± 3.18E-02 | 0.0001714 ± 1.98E-05 | 0.9919 |
| 10 | 0.2774 ± 5.02E-04 | 0.05889 ± 1.70E-04 | -0.2999 ± 2.24E-01 | 7.42E-05 ± 5.74E-05 | 0.9836 |

*^a^*The standard deviation values of fitting with equation 2 are shown.

# Table S3B. Slope values*^a^* of the SAH inhibition curves.

| [SAH], µM | a·k_1_, (s^-1^) | -b·k_2_, (s^-1^) |
| --- | --- | --- |
| 0 | 9.90E-03 ± 4.35E-05 | 6.71E-04 ± 1.55E-06 |
| 0.1 | 1.22E-02 ± 4.90E-05 | 6.77E-04 ± 1.35E-06 |
| 0.25 | 1.44E-02 ± 5.59E-05 | 5.61E-04 ± 1.30E-06 |
| 0.5 | 1.68E-02 ± 4.24E-05 | 3.52E-04 ± 1.25E-06 |
| 1 | 1.86E-02 ± 4.67E-05 | 2.34E-04 ± 1.38E-06 |
| 2.5 | 2.13E-02 ± 4.93E-05 | 1.10E-04 ± 2.16E-06 |
| 5 | 1.71E-02 ± 4.08E-05 | 5.14E-05 ± 8.08E-06 |
| 10 | 1.63E-02 ± 5.57E-05 | 2.22E-05 ± 2.71E-05 |

*^a^*The standard deviation of the slope values are calculated from the fitting standard deviation values of a, b, k_1_ and k_2_.

# Table S4A. Parameter values*^a^* of the sinefungin inhibition curves.

| [sinefungin], µM | a | k_1_, (s^-1^) | b | k_2_, (s^-1^) | R^2^ |
| --- | --- | --- | --- | --- | --- |
| 0 | 0.1624 ± 4.09E-04 | 0.06256 ± 2.85E-04 | -0.1869 ± 1.53E-04 | 3.88E-03 ± 7.52E-06 | 0.9970 |
| 0.0375 | 0.1645 ± 3.77E-04 | 0.0653 ± 2.62E-04 | -0.1699 ± 1.17E-04 | 3.38E-03 ± 6.86E-06 | 0.9975 |
| 0.075 | 0.2064 ± 3.83E-04 | 0.05564 ± 1.79E-04 | -0.2170 ± 1.94E-04 | 1.94E-03 ± 5.83E-06 | 0.9972 |
| 0.15 | 0.2215 ± 4.17E-04 | 0.05703 ± 1.83E-04 | -0.2165 ± 3.17E-04 | 1.62E-03 ± 6.43E-06 | 0.9964 |
| 0.3 | 0.2200 ± 4.51E-04 | 0.05374 ± 1.88E-04 | -0.1711 ± 6.89E-04 | 1.23E-03 ± 1.02E-05 | 0.9913 |
| 0.75 | 0.2668 ± 5.02E-04 | 0.05576 ± 1.77E-04 | -0.1045 ± 8.19E-04 | 1.18E-03 ± 1.83E-05 | 0.984 |
| 1.5 | 0.287 ± 5.51E-04 | 0.05722 ± 1.79E-04 | -0.1776 ± 8.27E-03 | 4.06E-04 ± 2.33E-05 | 0.9822 |
| 3 | 0.2925 ± 5.44E-04 | 0.05659 ± 1.69E-04 | -4.517 | 9.13E-06 ± 3.36E-05 | 0.9824 |
| 7.5 | 0.2481 ± 5.15E-04 | 0.05968 ± 1.97E-04 | -4.223 | 3.37E-06 ± 8.82E-05 | 0.979 |
| 15 | 0.2592 ± 5.43E-04 | 0.05599 ± 1.89E-04 | -0.5239 | 5.08E-06 ± 5.23E-04 | 0.9798 |

*^a^*The standard deviation values of fitting with equation 2 are shown.

# Table S4B. Slope values*^a^* of the sinefungin inhibition curves.

| [sinefungin], µM | a·k_1_, (s^-1^) | -b·k_2_, (s^-1^) |
| --- | --- | --- |
| 0 | 1.02E-02 ± 5.29E-05 | 7.24E-04 ± 1.53E-06 |
| 0.0375 | 1.07E-02 ± 4.96E-05 | 5.74E-04 ± 1.23E-06 |
| 0.075 | 1.15E-02 ± 4.27E-05 | 4.20E-04 ± 1.32E-06 |
| 0.15 | 1.26E-02 ± 4.69E-05 | 3.51E-04 ± 1.48E-06 |
| 0.3 | 1.18E-02 ± 4.79E-05 | 2.10E-04 ± 1.94E-06 |
| 0.75 | 1.49E-02 ± 5.48E-05 | 1.23E-04 ± 2.15E-06 |
| 1.5 | 1.64E-02 ± 6.01E-05 | 7.22E-05 ± 5.34E-06 |
| 3 | 1.66E-02 ± 5.83E-05 | 4.12E-05 ± 1.52E-04 |
| 7.5 | 1.48E-02 ± 5.77E-05 | 1.42E-05 ± 3.72E-04 |
| 15 | 1.45E-02 ± 5.77E-05 | 2.66E-06 ± 2.74E-04 |

*^a^*The standard deviation of the slope values are calculated from the fitting standard deviation values of a, b, k_1_ and k_2_.

# Table S5A. Parameter values*^a^* of the H4R3me2a inhibition curves.

| [H4R3Me2a], µM | a | k_1_, (s^-1^) | b | k_2_, (s^-1^) | R^2^ |
| --- | --- | --- | --- | --- | --- |
| 0 | 0.1800 ± 3.96E-04 | 0.06112 ± 2.52E-04 | -0.1961 ± 1.47E-04 | 0.003422 ± 7.36E-06 | 0.9967 |
| 0.025 | 0.1544 ± 3.91E-04 | 0.06982 ± 3.27E-04 | -0.1795 ± 1.47E-04 | 0.003946 ± 7.72E-06 | 0.9967 |
| 0.05 | 0.1589 ± 4.16E-04 | 0.06665 ± 3.21E-04 | -0.1863 ± 1.43E-04 | 0.003437 ± 7.65E-06 | 0.9963 |
| 0.1 | 0.1731 ± 3.91E-04 | 0.05356 ± 2.31E-04 | -0.1960 ± 1.50E-04 | 0.003104 ± 7.73E-06 | 0.9963 |
| 0.25 | 0.1692 ± 4.25E-04 | 0.05400 ± 2.60E-04 | -0.1883 ± 1.69E-04 | 0.003253 ± 8.83E-06 | 0.9953 |
| 0.5 | 0.1282 ± 4.32E-04 | 0.04557 ± 3.06E-04 | -0.1431 ± 2.10E-04 | 0.003370 ± 1.35E-05 | 0.9903 |
| 1 | 0.1156 ± 3.30E-04 | 0.0355 ± 2.01E-04 | -0.1396 ± 5.20E-04 | 0.001394 ± 1.31E-05 | 0.9878 |
| 2 | 0.0723 ± 3.03E-04 | 0.02475 ± 2.15E-04 | -0.07524 ± 5.65E-04 | 0.001365 ± 2.82E-05 | 0.9577 |

*^a^*The standard deviation values of fitting with equation 2 are shown.

# Table S5B. Slope values*^a^* of the H4R3me2a inhibition curves.

| [H4R3Me2a], µM | a·k_1_, (s^-1^) | -b·k_2_, (s^-1^) |
| --- | --- | --- |
| 0 | 1.10E-02 ± 5.14E-05 | 6.71E-04 ± 1.53E-06 |
| 0.025 | 1.08E-02 ± 5.74E-05 | 7.08E-04 ± 1.50E-06 |
| 0.05 | 1.06E-02 ± 5.80E-05 | 6.40E-04 ± 1.51E-06 |
| 0.1 | 9.27E-03 ± 4.52E-05 | 6.08E-04 ± 1.59E-06 |
| 0.25 | 9.14E-03 ± 4.96E-05 | 6.13E-04 ± 1.75E-06 |
| 0.5 | 5.84E-03 ± 4.38E-05 | 4.82E-04 ± 2.06E-06 |
| 1 | 4.10E-03 ± 2.60E-05 | 1.95E-04 ± 1.96E-06 |
| 2 | 1.79E-03 ± 1.72E-05 | 1.03E-04 ± 2.25E-06 |

*^a^*The standard deviation of the slope values are calculated from the fitting standard deviation values of a, b, k_1_ and k_2_.

# Table S6A. Parameter values*^a^* of the DB75 inhibition curves.

| [DB75], µM | a | k_1_, (s^-1^) | b | k_2_, (s^-1^) | R^2^ |
| --- | --- | --- | --- | --- | --- |
| 0 | 0.1445 ± 3.59E-04 | 0.06205 ± 2.82E-04 | -0.1741 ± 1.41E-04 | 0.004015 ± 7.30E-06 | 0.9973 |
| 2.5 | 0.1608 ± 3.86E-04 | 0.05913 ± 2.65E-04 | -0.203 ± 1.66E-04 | 0.004151 ± 7.18E-06 | 0.9975 |
| 5 | 0.1114 ± 3.10E-04 | 0.06653 ± 3.34E-04 | -0.14 ± 1.20E-04 | 0.004282 ± 7.68E-06 | 0.9971 |
| 7.5 | 0.1285 ± 3.57E-04 | 0.04791 ± 2.52E-04 | -0.1789 ± 1.33E-04 | 0.002865 ± 7.66E-06 | 0.9965 |
| 10 | 0.06466 ± 3.90E-04 | 0.03706 ± 4.47E-04 | -0.07628 ± 3.14E-04 | 0.004596 ± 2.82E-05 | 0.9754 |
| 20 | 0.02855 ± 5.16E-04 | 0.02992 ± 8.36E-04 | -0.01837 ± 5.38E-04 | 0.005985 ± 1.62E-04 | 0.6643 |
| 40 | 0.009937 ± 5.73E-04 | 0.1335 ± 1.03E-02 | - | - | 0.9104 |

*^a^*The standard deviation values of fitting with equation 2 are shown.

# Table S6B. Slope values*^a^* of the DB75 inhibition curves.

| [DB75], µM | a·k_1_, (s^-1^) | -b·k_2_, (s^-1^) |
| --- | --- | --- |
| 0 | 8.97E-03 ± 4.64E-05 | 6.990E-04 ± 1.39E-06 |
| 2.5 | 9.51E-03 ± 4.83E-05 | 8.427E-04 ± 1.61E-06 |
| 5 | 7.41E-03 ± 4.25E-05 | 5.995E-04 ± 1.19E-06 |
| 7.5 | 6.16E-03 ± 3.66E-05 | 5.125E-04 ± 1.42E-06 |
| 10 | 2.40E-03 ± 3.23E-05 | 3.506E-04 ± 2.59E-06 |
| 20 | 8.54E-04 ± 2.84E-05 | 1.10E-04 ± 4.39E-06 |
| 40 | 1.33E-03 ± 1.28E-04 | 0 |

*^a^*The standard deviation of the slope values are calculated from the fitting standard deviation values of a, b, k_1_ and k_2_.

# Table S7A. Parameter values*^a^* of the MS023 inhibition curves.

| [MS023], µM | a | k_1_, (s^-1^) | b | k_2_, (s^-1^) | R^2^ |
| --- | --- | --- | --- | --- | --- |
| **0** | 0.1306 ± 3.23E-04 | 0.06923 ± 3.11E-04 | -0.1598 ± 1.36E-04 | 0.004868 ± 7.47E-06 | 0.9976 |
| **0.01** | 0.1705 ± 4.53E-04 | 0.05716 ± 2.73E-04 | -0.2028 ± 1.46E-04 | 0.002997 ± 7.44E-06 | 0.9964 |
| **0.02** | 0.1706 ± 3.73E-04 | 0.05384 ± 2.22E-04 | -0.1507 ± 1.57E-04 | 0.003684 ± 9.45E-06 | 0.9951 |
| **0.05** | 0.1741 ± 5.03E-04 | 0.05062 ± 2.70E-04 | -0.1701 ± 1.74E-04 | 0.002741 ± 1.07E-05 | 0.9924 |
| **0.1** | 0.1833 ± 4.85E-04 | 0.04454 ± 2.11E-04 | -0.1468 ± 1.36E-03 | 0.0010 ± 1.66E-05 | 0.9797 |
| **0.2** | 0.1330 ± 2.05E-03 | 0.03397 ± 3.62E-04 | - | - | 0.9648 |

*^a^*The standard deviation values of fitting with equation 2 are shown.

# Table S7B. Slope values*^a^* of the MS023 inhibition curves.

| **[MS023], µM** | **a·k_1_, (s^-1^)** | **-b·k_2_, (s^-1^)** |
| --- | --- | --- |
| 0 | 9.04E-03 ± 4.64E-05 | 7.78E-04 ± 1.36E-06 |
| 0.01 | 9.75E-03 ± 5.33E-05 | 6.08E-04 ± 1.57E-06 |
| 0.02 | 9.19E-03 ± 4.29E-05 | 5.55E-04 ± 1.54E-06 |
| 0.05 | 8.81E-03 ± 5.35E-05 | 4.66E-04 ± 1.88E-06 |
| 0.1 | 8.16E-03 ± 4.42E-05 | 1.47E-04 ± 2.79E-06 |
| 0.2 | 4.52E-03 ± 8.45E-05 | - |

*^a^*The standard deviation of the slope values are calculated from the fitting standard deviation values of a, b, k_1_ and k_2_.


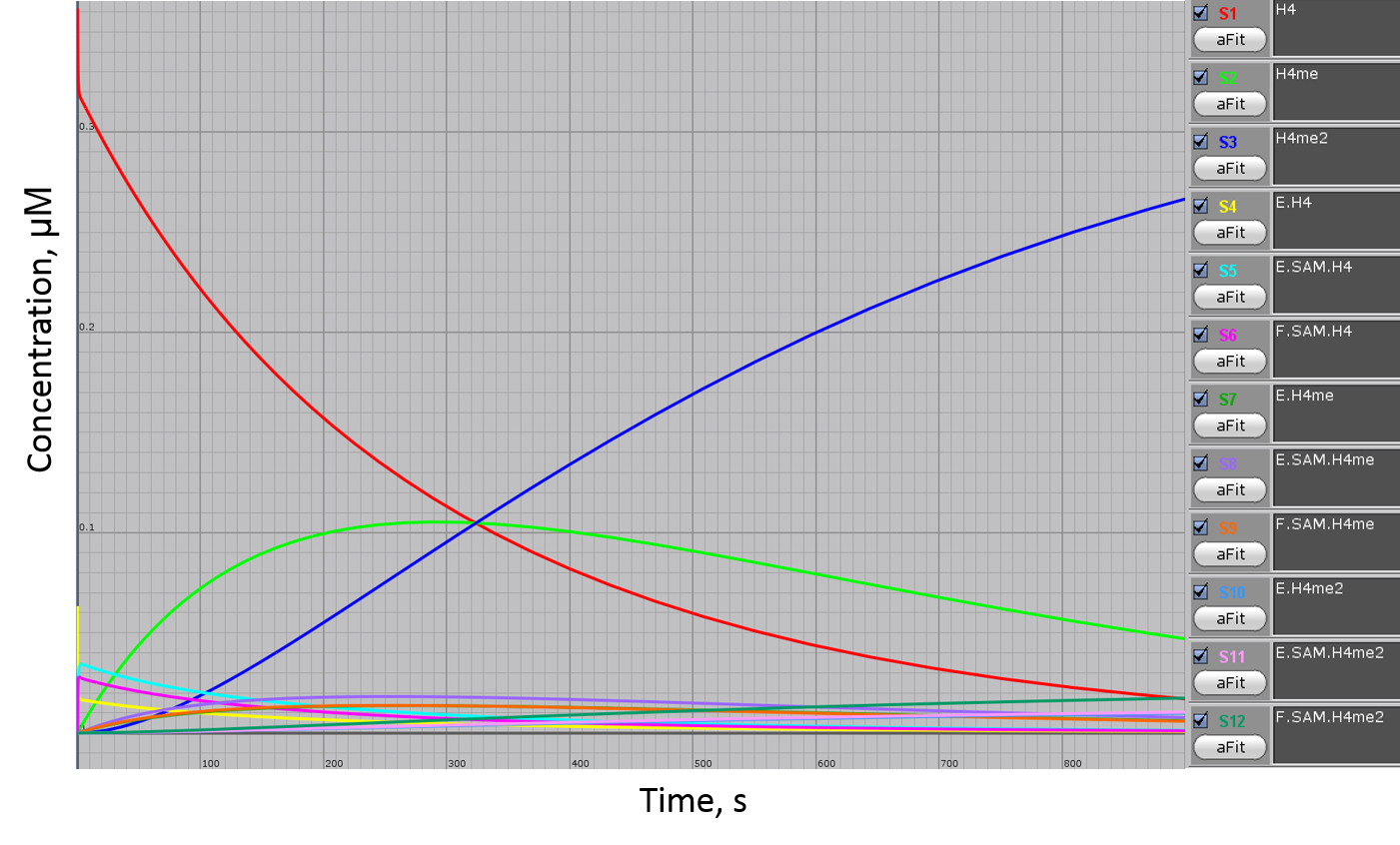


# Figure S1A. Concentration changes of peptide-related species during the reaction ([PRMT1] = 0.2 µM, [SAM] = 3.5 µM, [H4] = 0.4 µM), simulated based on the complete kinetics model of PRMT1^1^ using KinTek Explorer 5.2.


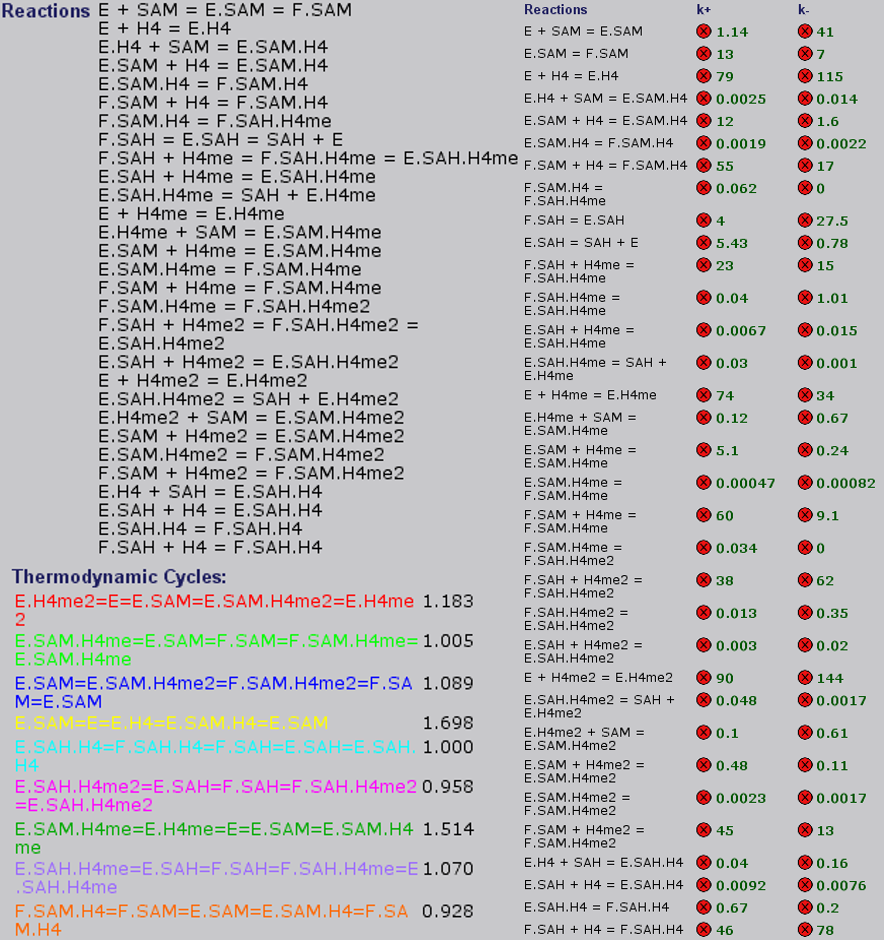


# Figure S1B. The parameter values used for the simulation of Figure S1A.


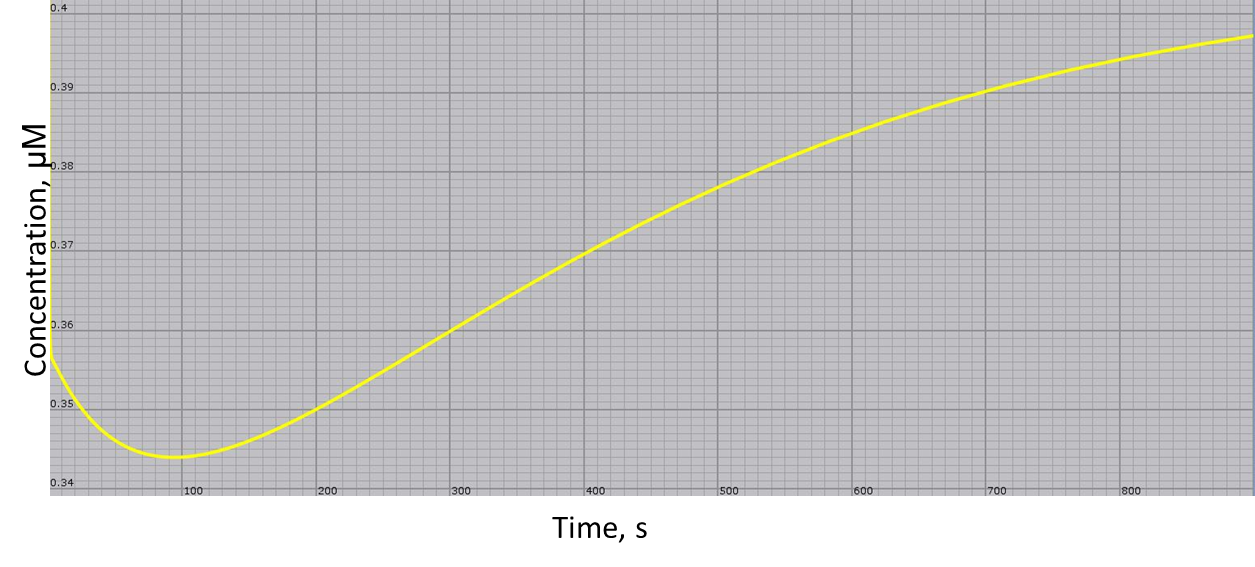


**Figure S1C.** Total concentration curve of the free peptides ([H4FL] + [H4FLme] + [H4FLme2]), based on the simulation result of **Figure S1A**.


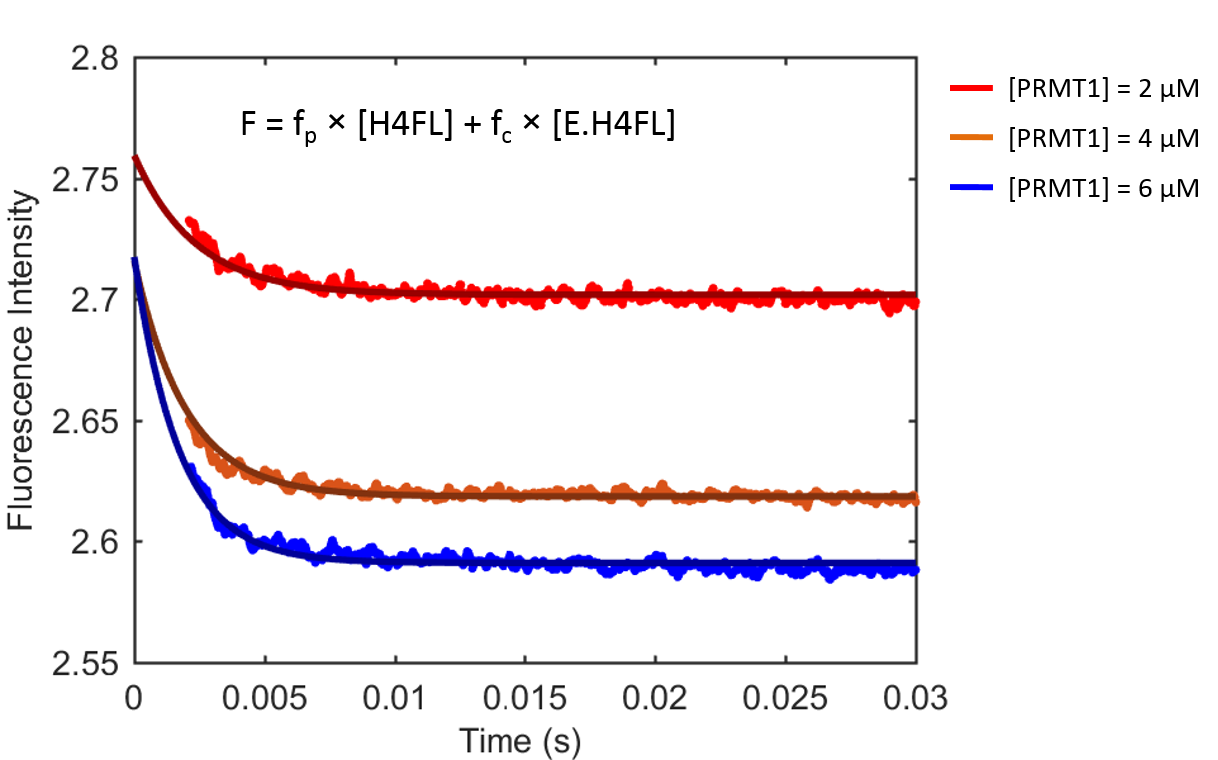


# Figure S2. Global fitting result based on the binary binding model of PRMT1 and H4FL. The values of k_on_ and k_off_ 40 µM^-1^s^-1^ and 333 s^-1^ were used^2^, respectively. The resulted value of fp and fc are 6.4 µM^-1^ and 5.6 µM^-1^, respectively. The raw data are shown in colored points and the smooth lines are the simulation results. H4FL concentration is fixed at 0.4 µM.

**
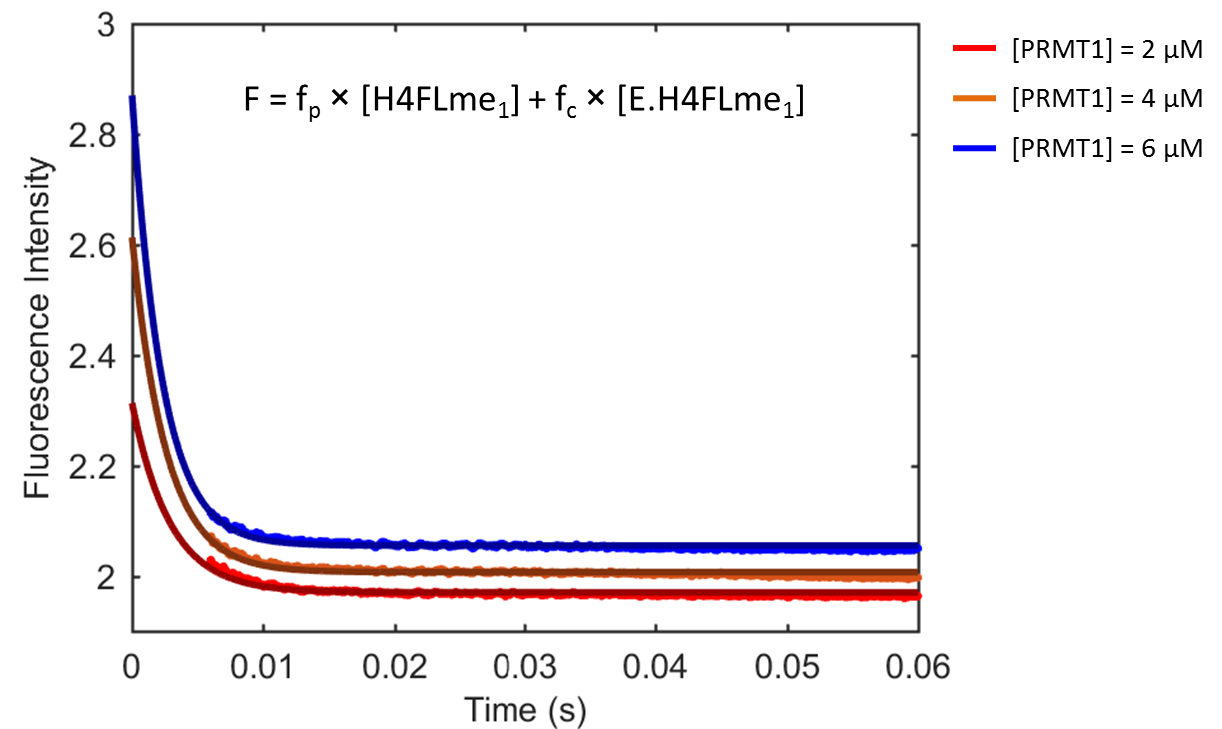
**

# Figure S3. Global fitting results based on the binary binding model of PRMT1 and H4meFL. The values of k_on_ and k_off_ of 23 µM^-1^s^-1^ and 292 s^-1^ were used^2^, respectively. The resulted value of f_p_ and f_c_ are 5.4 µM^-1^ and 3.8 µM^-1^, respectively. The raw data are shown in colored points and the smooth lines are the simulation results. H4FLme_1_ concentration is fixed at 0.4 µM.

**
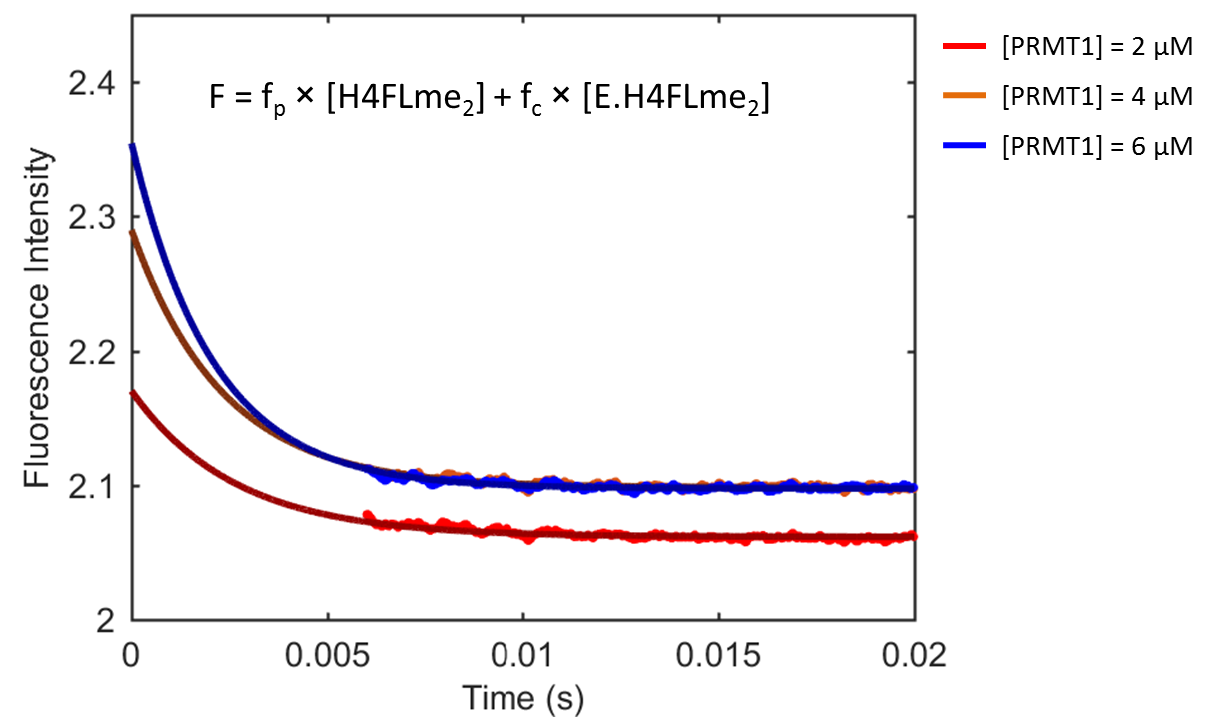
**

# Figure S4. Global fitting results based on the binary binding model of PRMT1 and H4me_2a_FL. The values of k_on_ and k_off_ of 26 µM^-1^s^-1^ and 319 s^-1^ were used^2^, respectively. The resulted value of f_p_ and f_c_ are 5.9 µM^-1^ and 3.9 µM^-1^, respectively. The raw data are shown in colored points and the smooth lines are the simulation results. H4FLme_2_ concentration is fixed at 0.4 µM.


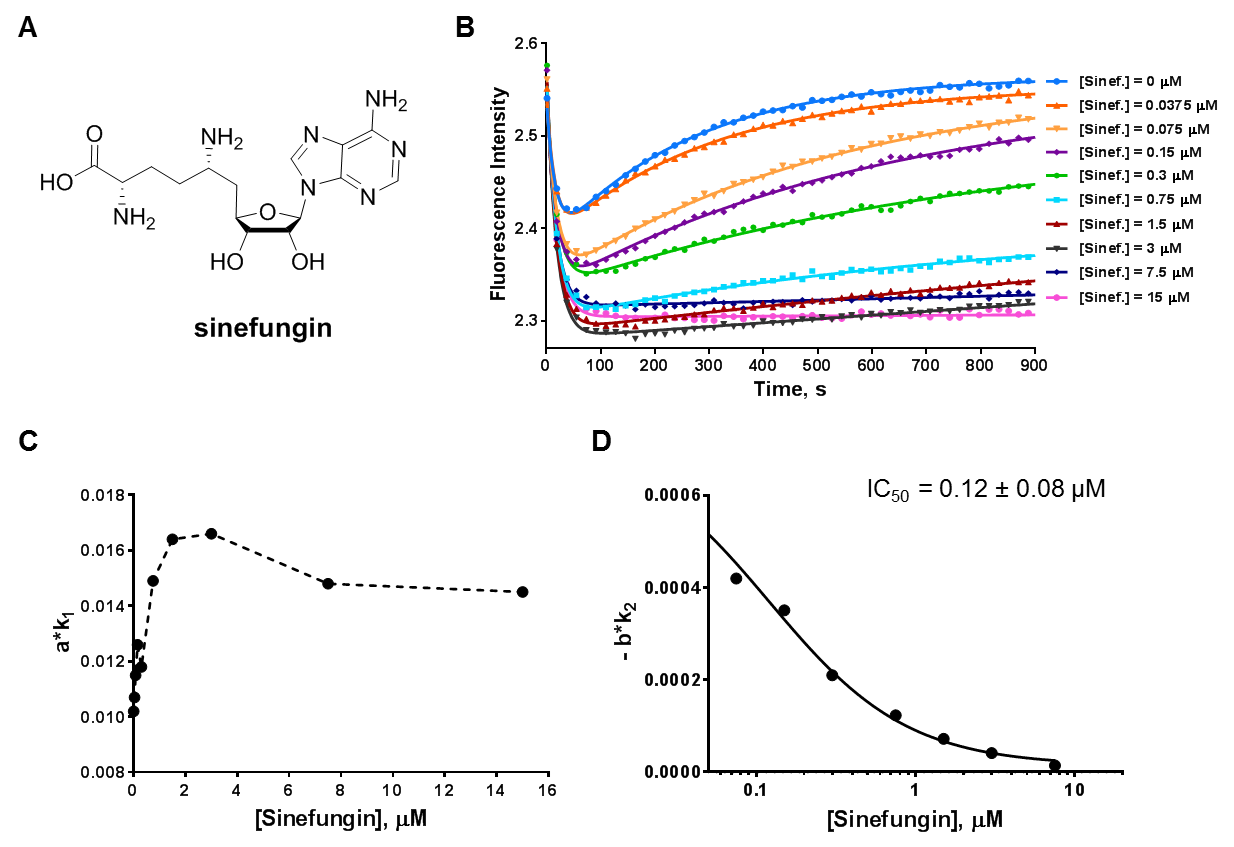


# Figure S5. Stopped-flow fluorescence assay of the cofactor competitive inhibitor, sinefungin. A. Structure of sinefungin. In B, the curves are fit with equation 2 by Prism to generate values in Table 4A. Each curve used 10,000 data points, but only 50 data points are shown. Each curve is an average of 4 or 5 replicates. C and D represent the relationship of a·k_1_ or b·k_2_ with inhibitor concentrations, values listed in Table 4B. In D, the IC_50_ is calculated using equation 1. The reaction condition used for all experiments are [PRMT1] = 0.2 µM, [SAM] = 3.5 µM, [H4FL] = 0.4 µM, with varying concentrations of sinefungin.


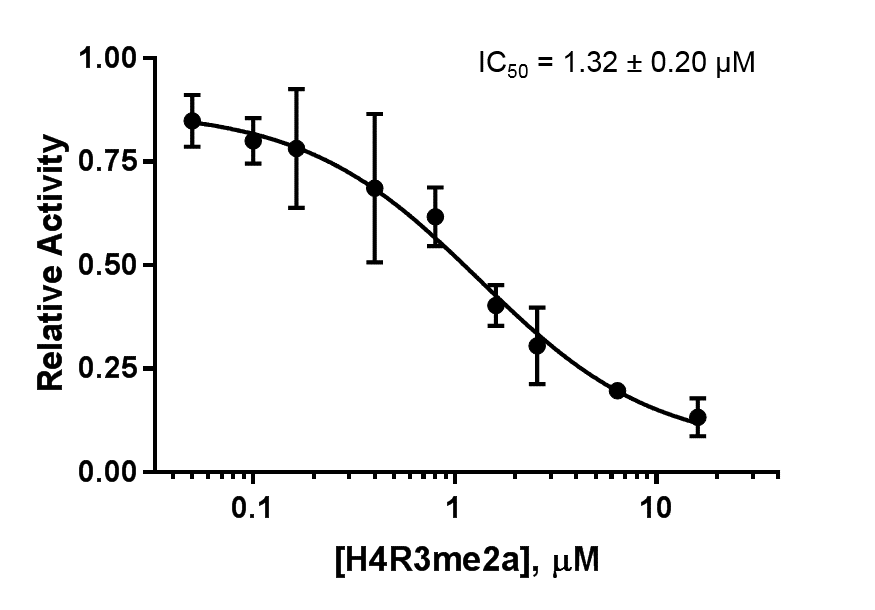


# Figure S6. IC_50_ of H4R3Me_2a_ by filter binding assay, at the following condition: [PRMT1] = 0.2 µM, [SAM] = 3.5 µM, [H4FL] = 0.4 µM, with varying concentration of H4R3Me_2a_.


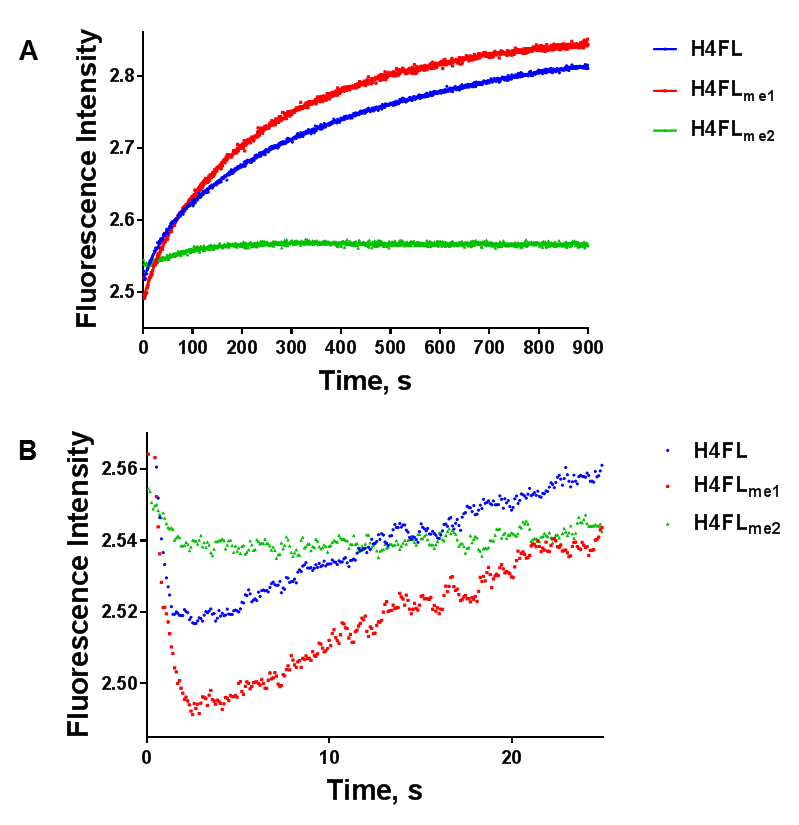


# Figure S7. Stopped-flow fluorescence assay of H4FL, H4FLme_1_ and H4FLme_2_ peptides. A. Reaction time curves of H4FL, H4FLme_1_ and H4FLme_2_ up to 900s. For each curve, 10,000 data points were obtained and shown. Each curve is an average of 4 or 5 replicates. B. An enlarged view of A for up to 25 s. The reaction conditions used for all experiments are [PRMT1] = 0.2 µM, [SAM] = 3.5 µM, and [H4 peptides] = 0.4 µM.

**References**

1 Hu, H.; Luo, C.; Zheng, Y. G., Transient Kinetics Define a Complete Kinetic Model for Protein Arginine Methyltransferase 1. *J. Biol. Chem.* **2016,** *291* (52), 26722-26738.

2 Feng Y, Xie N, Jin M, Stahley MR, Stivers JT, Zheng YG. A transient kinetic analysis of PRMT1 catalysis. Biochemistry 2011; 50: 7033-7044.
